# Prospective SARS-CoV-2 additional vaccination in immunosuppressant-treated individuals with autoimmune diseases in a randomized controlled trial

**DOI:** 10.1172/jci.insight.191266

**Published:** 2025-11-25

**Authors:** Meggan Mackay, Catriona A. Wagner, Ashley Pinckney, Jeffrey A. Cohen, Zachary S. Wallace, Arezou Khosroshahi, Jeffrey A. Sparks, Sandra Lord, Amit Saxena, Roberto Caricchio, Alfred H.J. Kim, Diane L. Kamen, Fotios Koumpouras, Anca D. Askanase, Kenneth Smith, Joel M. Guthridge, Gabriel Pardo, Yang Mao-Draayer, Susan Macwana, Sean McCarthy, Matthew A. Sherman, Sanaz Daneshfar Hamrah, Maria Veri, Sarah Walker, Kate York, Sara K. Tedeschi, Jennifer Wang, Gabrielle Dziubla, Mike Castro, Robin Carroll, Sandeep Narpala, Bob C. Lin, Leonid Serebryannyy, Adrian B. McDermott, ACV01 Study Team, William T. Barry, Ellen Goldmuntz, James McNamara, Aimee S. Payne, Amit Bar-Or, Dinesh Khanna, Judith A. James

**Affiliations:** 1 Institute of Molecular Medicine, Feinstein Institutes for Medical Research, Manhasset, New York, USA.; 2 Arthritis and Clinical Immunology, Oklahoma Medical Research Foundation, Oklahoma City, Oklahoma, USA.; 3 Rho, Durham, NC, USA.; 4 Neurologic Institute, Cleveland Clinic, Cleveland, Ohio, USA.; 5 Division of Rheumatology, Allergy, and Immunology, Massachusetts General Hospital, Boston, Massachusetts, USA.; 6 Harvard Medical School, Boston, Massachusetts, USA.; 7 Emory University, Atlanta, Georgia, USA.; 8 Division of Rheumatology, Inflammation, and Immunity, Brigham and Women’s Hospital, Boston, Massachusetts, USA.; 9 Center for Interventional Immunology, Benaroya Research Institute, Seattle, Washington, USA,; 10 Division of Rheumatology, Department of Medicine, New York University, New York, USA.; 11 Division of Rheumatology, UMass Chan Medical School, Worcester, Massachusetts, USA.; 12 Division of Rheumatology, Department of Medicine, Washington University School of Medicine, St. Louis, Missouri, USA.; 13 Medical University of South Carolina, Charleston, South Carolina, USA.; 14 Department of Internal Medicine, Section of Rheumatology, Allergy & Immunology, Yale School of Medicine, New Haven, Connecticut, USA.; 15 Department of Medicine, Columbia University Irving Medical Center, New York, New York, USA.; 16 Department of Neurology, University of Michigan, Ann Arbor, Michigan, USA.; 17 Division of Allergy, Immunology, and Transplantation, NIH/NIAID, Bethesda, Maryland, USA.; 18 Vaccine Research Center, NIH/NIAID, Bethesda, Maryland, USA.; 19 The ACV01 Study Team members (detailed in Supplemental Acknowledgments).; 20 Department of Dermatology, Columbia University, New York, New York, USA.; 21 Department of Neurology, Perelman School of Medicine, University of Pennsylvania, Philadelphia, Pennsylvania, USA.; 22 Division of Rheumatology, Department of Internal Medicine, University of Michigan, Ann Arbor, Michigan, USA.; 23 Departments of Medicine and Pathology, University of Oklahoma Health Science Center, Oklahoma City, Oklahoma, USA.

**Keywords:** Autoimmunity, COVID-19, Vaccines, Autoimmune diseases, Clinical practice, Rheumatology

## Abstract

**BACKGROUND:**

Individuals with autoimmune diseases (ADs) on immunosuppressants often have suboptimal responses to COVID-19 vaccines. We evaluated the efficacy and safety of additional COVID-19 vaccines in those treated with mycophenolate mofetil/mycophenolic acid (MMF/MPA), methotrexate (MTX), and B cell–depleting therapy (BCDT), including the impact of withholding MMF/MPA and MTX.

**METHODS:**

In this open-label, multicenter, randomized trial, 22 participants taking MMF/MPA, 26 taking MTX, and 93 treated with BCDT who had suboptimal antibody responses to initial COVID-19 vaccines (2 doses of BNT162b2 or mRNA-1273 or 1 dose of AD26.COV2.S) received an additional homologous vaccine. Participants taking MMF/MPA and MTX were randomized (1:1) to continue or withhold treatment around vaccination. The primary outcome was the change in anti–Wuhan-Hu-1 receptor-binding domain (RBD) concentrations at 4 weeks after additional vaccination. Secondary outcomes included adverse events, COVID-19, and AD activity through 48 weeks.

**RESULTS:**

Additional vaccination increased anti-RBD concentrations in participants taking MMF/MPA and MTX, irrespective of immunosuppressant withholding. BCDT-treated participants also demonstrated increased anti-RBD concentrations, albeit lower than MMF/MPA- and MTX-treated cohorts. COVID-19 occurred in 33% of participants; infections were predominantly mild and included only 3 nonfatal hospitalizations. Additional vaccination was well tolerated, with low frequencies of severe disease flares and adverse events.

**CONCLUSION:**

Additional COVID-19 vaccination is effective and safe in individuals with ADs treated with immunosuppressants, regardless of whether MMF/MPA or MTX is withheld.

**TRIAL REGISTRATION:**

ClinicalTrials.gov (NCT05000216; registered August 6, 2021: https://clinicaltrials.gov/ct2/show/NCT05000216).

**FUNDING:**

The NIH/NIAID supported the study through the Autoimmunity Centers of Excellence and the Intramural Research Program

## Abstract 2

In a clinical trial, additional COVID-19 vaccination was effective and safe in autoimmune patients on immunosuppressants, increasing antibody levels regardless of treatment continuation.

## Introduction

Individuals with autoimmune diseases (ADs) treated with immunosuppressive (IS) drugs, such as methotrexate (MTX), mycophenolate mofetil/mycophenolic acid (MMF/MPA), and B cell–depleting therapies (BCDTs), exhibit impaired humoral responses following initial COVID-19 vaccination series, which is associated with an increased risk of breakthrough infections (1–3). Therefore, improving vaccine efficacy in individuals with an inadequate humoral immune response is crucial. In BCDT- and MMF/MPA-treated individuals with ADs, additional vaccination induces a humoral response in some seronegative individuals, but seropositivity remains at a lower frequency and lower levels compared with controls (4–7). In MTX-treated individuals with ADs, seroconversion is relatively high following the primary vaccine series; however, antibody levels are decreased compared with controls (8). While an additional vaccination further increases anti–SARS-CoV-2 antibody levels, conflicting data exist on whether antibody levels remain lower compared with controls (5, 7, 9–12). Furthermore, prospective clinical trial data on the effects of additional vaccination on breakthrough infections in individuals treated with MTX, MMF/MPA, and BCDT are lacking, as are clinical trials addressing the immunogenicity of additional vaccination in these populations.

MMF/MPA and MTX can impair vaccine responses, and current guidelines recommend withholding MMF/MPA and MTX for 1–2 weeks around each COVID-19 vaccine dose (13). However, there are limited data from clinical trials demonstrating the benefit and safety of this approach following additional vaccination. In the VROOM clinical trial, a 2-week hold of MTX following a third vaccination with BNT162b2 (Pfizer-BioNTech), mRNA-1273 (Moderna), or AZD1222 (AstraZeneca) vaccines in individuals with immune-mediated inflammatory disease resulted in a 2.19-fold increase in antibodies against the receptor-binding domain (RBD) compared with individuals who continued MTX (14). This response was sustained through 26 weeks after the third vaccination (15). Although withholding MTX temporarily increased self-reported disease flares, the majority of flares were limited, self-managed, and did not require medical attention, with no serious adverse events (SAEs) or deaths (14, 15). Despite improved humoral responses, new COVID-19 diagnosis throughout the VROOM trial occurred at similar frequencies and severity regardless of MTX suspension (15). While less is known about the effects of MMF/MPA withdrawal on SARS-CoV-2 vaccine responses in individuals with ADs, an observational study of 195 individuals with rheumatic and musculoskeletal diseases demonstrated that withholding MMF/MPA near the time of vaccination increased the frequency and titer of anti-RBD antibodies (16). Indeed, limited clinical trial data support current recommendations regarding the withholding of IS treatment, especially regarding clinical vaccine efficacy and the effects in individuals with inadequate or modest humoral responses to the primary vaccine series.

In this randomized, multisite, open-label clinical trial, we determined the immunogenicity, safety, and clinical efficacy of a homologous additional COVID-19 vaccine dose in individuals with ADs on MTX, MMF/MPA, or BCDT with suboptimal responses to the primary COVID-19 vaccine series. In addition, we evaluated the impacts of temporarily withholding MTX or MMF/MPA around the time of vaccination on the vaccine response.

## Results

### Study participants.

**Figure 1:**
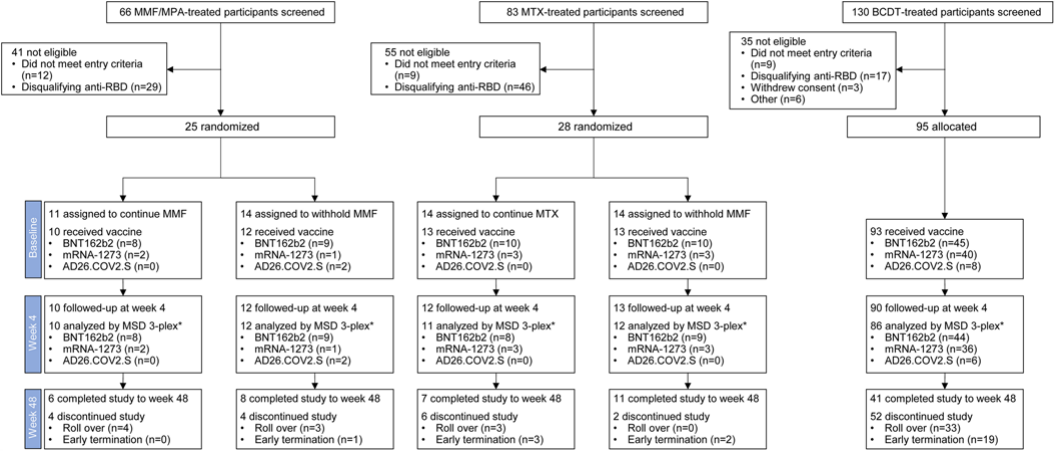
ACV01 trial profile. MMF, mycophenolate mofetil; MPA, mycophenolic acid; MTX, methotrexate; BCDT, B cell–depleting therapy. *Samples were excluded from the humoral response analyses if the participant experienced COVID-19, monoclonal antibody use, or a COVID-19 vaccination off-study between the baseline and week 4 visit. Some individuals were rolled over to Stage 2 of this study to assess a booster with a nonhomologous vaccine; Stage 2 results are not reported here.

Between August 2021 and August 2022, we screened 279 participants for Stage 1, of whom 148 were allocated to 3 cohorts based on their current treatment regimen: MMF/MPA (*n* = 25), MTX (*n* = 28), and BCDT (*n* = 95; Figure 1 and Supplemental Figure 1; supplemental material available online with this article; https://doi.org/10.1172/jci.insight.191266DS1). Participants in the MMF/MPA and MTX cohorts were randomized to continue or withhold treatment around the time of additional vaccination; 11 in the MMF/MPA cohort continued and 14 withheld, 14 in the MTX cohort continued, and 14 withheld (Figure 1 and Supplemental Figure 1). At baseline, 140 participants received an additional vaccination, defined as a third dose of BNT162b2 (*n* = 82) or mRNA-1273 (*n* = 48) or a second dose of AD26.COV2.S (Janssen) (*n* = 10). Due to the small sample sizes, participants who received either BNT162b2 (Pfizer-BioNTech) or mRNA-1273 (Moderna) were analyzed together, while those who received the AD26.COV2.S vaccine were analyzed separately.

Baseline demographics were similar between cohorts in the vaccinated population. Most participants were women (MMF/MPA, 91%; MTX, 96%; BCDT, 71%), with the majority identifying as White or Black and non-Hispanic or Latino (Table 1). The median age for all cohorts was 54 years. The MMF/MPA cohort consisted of 73% (*n* = 16) participants with systemic lupus erythematosus (SLE), 23% (*n* = 5) with systemic sclerosis (SSc), and 5% (*n* = 1) with pemphigus, while the MTX cohort consisted of 77% (*n* = 20) participants with rheumatoid arthritis (RA) and 23% (*n* = 6) with SLE (Table 1). Most participants in the BCDT cohort had multiple sclerosis (MS) (72%, *n* = 67) and RA (19%, *n* = 18). Only 1 (5%) MMF/MPA-treated and 7 (8%) BCDT-treated participants had a prior COVID-19 diagnosis as self-reported at screening. Results form the Roche Elecsys Anti-SARS-CoV-2 S assays done at screening demonstrated that few participants in the MMF/MPA- (*n* = 9; 41%) and MTX-treated (*n* = 1; 4%) cohorts exhibited a negative humoral response (<0.79 U/mL) to the initial COVID-19 vaccination, while the majority in the BCDT cohort (*n* = 69; 74%) had a negative humoral response (Table 1). Most participants taking MMF/MPA had SLE, with mean doses of 2375 ± 806.2 mg daily for MMF and 900 ± 763.7 mg daily for MPA. Most participants taking MTX had RA, with a mean dose of 17 ± 5.6 mg weekly (Supplemental Table 1). The majority of participants with MS were treated with ocrelizumab (*n* = 62), and a few were on ofatumumab (*n* = 3). Most of the participants receiving rituximab had RA (*n* = 18). The mean steroid dose was 4–6 mg daily across the participants receiving prednisone (SLE, *n* = 14; RA, *n* = 6; pemphigus, *n* = 1) (Supplemental Table 1).

### A third mRNA vaccine dose increases the humoral response against Wuhan-Hu-1 RBD and spike proteins, regardless of withholding medications.

**Figure 2:**
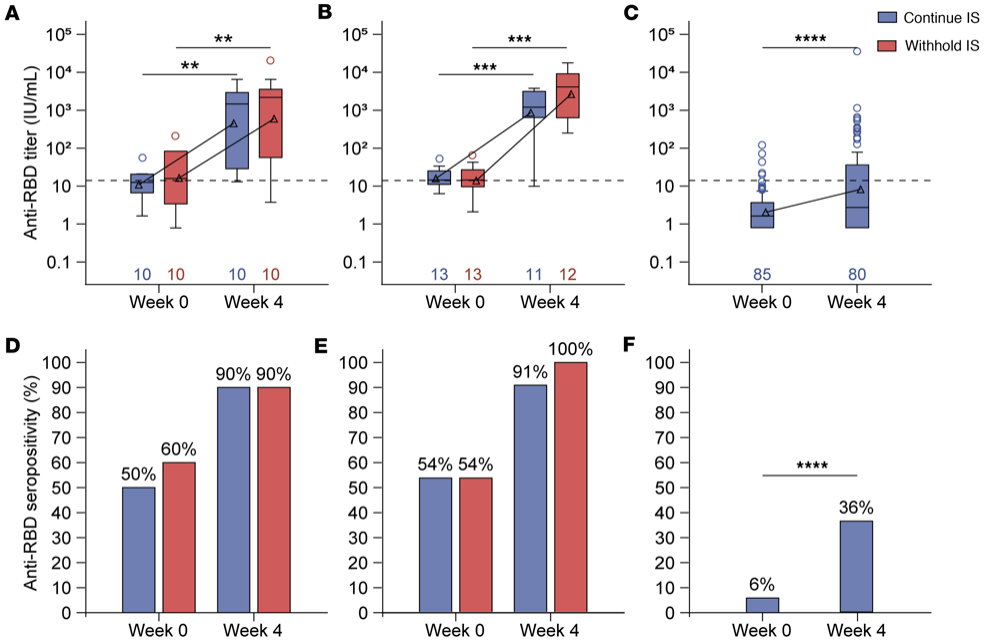
Anti-RBD concentrations and seropositivity in participants treated with mycophenolate mofetil/mycophenolic acid (MMF/MPA), methotrexate (MTX), or B cell–depleting therapy (BCDT) who received a third mRNA vaccine. Concentrations of anti-RBD antibodies at baseline and 4 weeks after the third vaccination in participants in the (**A**) MMF/MPA, (**B**) MTX, or (**C**) BCDT cohorts. Box-and-whisker plots display the interquartile range (IQR), with the line within the box indicating median values. Geometric mean values are represented by triangles and connected across study visits. Whiskers extend to 1.5 × IQR, with circles indicating outliers. The dashed line represents the positivity cutoff, and numbers indicate the number of participants analyzed at each time point. The Wilcoxon signed-rank test was used to assess the change in antibody concentration from baseline to week 4 within each group. There was no significant difference in concentrations between those who continued or withheld immunosuppressants, as determined by van Elteren’s test. Seropositivity of anti-RBD antibodies at baseline and 4 weeks after the third vaccination in participants in the (**D**) MMF/MPA, (**E**) MTX, or (**F**) BCDT cohorts. Statistical significance was determined using McNemar’s test. **P < 0.01; ***P < 0.001; ****P < 0.0001.

Concentrations of antibodies against Wuhan-Hu-1 RBD were significantly higher at 4 weeks after the third mRNA vaccination compared with baseline in all cohorts (Figure 2, A–C. While increases in the humoral response did not differ statistically between participants who withheld their medication and those who did not in both the MMF/MPA (*P* = 0.79) and MTX cohorts (*P* = 0.16), there was a numerical trend toward higher geometric mean concentrations in participants who withheld therapy (Supplemental Table 2). At baseline, approximately half of the participants who received an mRNA vaccine were seropositive for Wuhan-Hu-1 RBD in MMF/MPA- (continue, *n* = 5 [50%]; withhold, *n* = 6 [60%]) and MTX-treated (continue, *n* = 7 [54%]; withhold, *n* = 7 [54%]) cohorts (Figure 2, D and E). A third mRNA vaccine dose increased seropositivity to 90% (*n* = 9) and 91% (*n* = 10) in participants who continued MMF/MPA or MTX, respectively, and 90% (*n* = 9) and 100% (*n* = 12) in those who withheld MMF/MPA or MTX, respectively; however, the increase in seropositivity from baseline to 4 weeks in each group was not statistically significant (Figure 2, D and E). Although a third mRNA vaccine dose increased the humoral response in the BCDT-treated cohort from 6% (*n* = 5) to 36% (*n* = 29; *P* < 0.0001) at 4 weeks, seropositivity was still lower compared with the MMF/MPA- and MTX-treated participants (Figure 2F). Similar humoral response increases were observed against the Wuhan-Hu-1 spike protein and Roche Elecsys anti-RBD and in participants who received the AD26.COV2.S vaccine (Supplemental Figures 2–4 and Supplemental Table 2). Humoral responses appeared sustained in the subgroups that remained in the study through 48 weeks, regardless of cohort and treatment withholding (Supplemental Figure 5).

### Antibody response and neutralization across SARS-CoV-2 variants.

Next, we examined the effects of a third mRNA vaccine dose on anti-spike antibody concentrations across SARS-CoV-2 variants. Across all cohorts, concentrations of Wuhan-Hu-1 anti-spike antibodies were comparable to those against the Alpha, Beta, and Delta strains (Supplemental Figure 6, A and B, and Supplemental Table 3). Consistent with the general population (17), participants exhibited lower humoral responses against Omicron spike variants compared with the Wuhan-Hu-1 spike at baseline and 4 weeks after the third mRNA vaccination (Supplemental Figure 6, C and D, and Supplemental Table 3). Anti-RBD responses to the Wuhan-Hu-1 variant at week 4 after the third mRNA vaccination correlated strongly with Wuhan-Hu-1 live virus neutralization titers (*r* = 0.86; *P* < 0.0001) (Supplemental Figure 7), and fold inhibition of ACE2 binding was similar for the Wuhan-Hu-1, Alpha, Beta, and Delta SARS-CoV-2 strains (Supplemental Figure 8A). However, ACE2 neutralization was significantly reduced against Omicron subvariants BA.1, BA.2, BA.2.12.1, BA.2.75, and BA.5 across all cohorts (Supplemental Figure 8B). Neutralizing activity showed numerical trends toward higher ACE2 fold inhibition in participants who withheld therapy, with the most pronounced differences observed in the MTX cohort, while BCDT patients demonstrated uniformly low neutralizing activity regardless of variant (Supplemental Table 4).

### Disease type and baseline CD19^+^ cell count are associated with subsequent anti-RBD response.

In MMF/MPA- or MTX-treated participants who received an mRNA vaccine, individuals with RA had the highest anti-RBD concentrations at week 4 after vaccination compared with those with SLE, SSc, and pemphigus (Supplemental Table 5). In BCDT-treated participants who received an mRNA vaccine, detectable baseline B cell counts, but not the time from the last BCDT dose, was associated with higher anti-RBD concentrations (Supplemental Table 5). Week 4 humoral responses were not associated with the type of mRNA vaccine, medication withholding, sex, age, time since the initial COVID-19 vaccination, prior COVID-19, prednisone use, or type of medication in either cohort.

### COVID-19 is mild following a third mRNA vaccine dose.

Consistent with a low frequency of self-reported infection before additional vaccination (Table 1), seropositivity and titers of antibodies against Wuhan-Hu-1 nucleocapsid (N) were low at baseline and did not increase 4 weeks after the third mRNA vaccination in participants who received an mRNA vaccine (Supplemental Figure 9). COVID-19 occurred at a median of 62–110 days, with infections occurring in 33% of participants during study participation, with similar frequencies across treatment cohorts (Table 2 and Supplemental Figure 10). All infections (*n* = 7) were deemed mild in the MMF/MPA cohort, but most (6 of 10) were moderate in the MTX cohort (Table 2). Approximately half of the infections were mild in the BCDT cohort. Three hospitalizations occurred (1 in the withhold MTX- and 2 in the BCDT-treated group; Table 2), and none were life-threatening or fatal (grade 4 or 5). Infections were observed across the diseases studied and IS therapies (Table 2).

### A third mRNA vaccine dose is safe in individuals with ADs and does not affect disease activity.

Within 7 days of additional vaccination, local and systemic reactions were predominantly mild to moderate across all cohorts and vaccines (Supplemental Figure 11). Throughout the study, 39%–46% of participants who received an mRNA vaccine reported AEs, 6 of which (all BCDT-treated) were related to the vaccine (Supplemental Table 6). Furthermore, most AEs were grade 1 or 2, with 1 participant (8%) in the withhold MTX cohort and 9 participants (11%) in the BCDT-treated cohort experiencing an SAE, of which all were grade 3, and only 1 was categorized as vaccine-related (Supplemental Table 6). No grade 4 or grade 5 AEs occurred. In the BCDT-treated cohort, 1 (1%) participant experienced a medically attended AE (MAAE), and 7 participants (8%) experienced a new-onset chronic medical condition (NOCMC), none of which was a new autoimmune diagnosis (Supplemental Table 6). No participants experienced myocarditis or pericarditis during the study period (Supplemental Table 6). Participants who received the AD26.COV2.S vaccine did not experience vaccine-related AEs (Supplemental Table 7).

**Figure 3:**
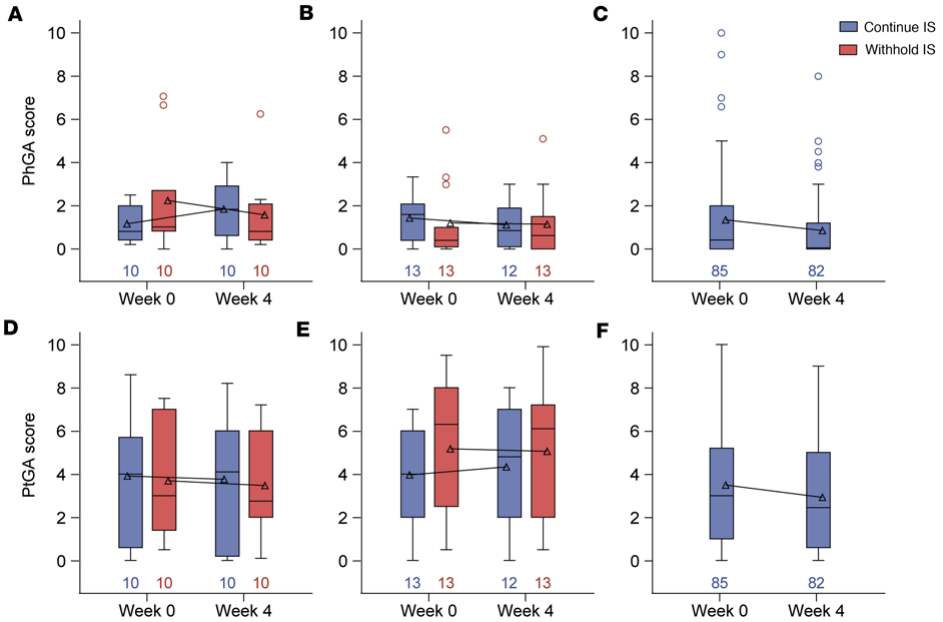
Disease activity in the vaccinated population that received a third mRNA vaccine. Changes in disease activity as measured by the (**A**–**C**) Physician Global Assessment (PGA) and (**D**–**F**) Patient Global Assessment (PtGA) at baseline and week 4 after booster in participants in the (**A** and **D**) mycophenolate mofetil/mycophenolic acid (MMF/MPA), (**B** and **E**) methotrexate (MTX), or (**C** and **F**) B cell–depleting therapy (BCDT) cohorts. Box-and-whisker plots display the interquartile range (IQR), with the line within the box indicating median values. Geometric mean values are represented by triangles and connected across study visits. Whiskers extend to 1.5 × IQR, with circles indicating outliers. The dashed line represents the positivity cutoff, and numbers indicate the number of participants analyzed at each time point. Change in score from baseline to week 4 did not significantly differ, as determined by the Wilcoxon signed-rank test.

In all cohorts, the Physician’s Global Assessment and Patient’s Global Assessment of disease activity were similar at baseline and 4 weeks after the third mRNA vaccine dose, regardless of whether medications were withheld (Figure 3). Similarly, most participants had no change based on the Clinical Global Impression of Change and Patient Global Impression of Change scales 4 weeks after the third mRNA vaccination, with some participants who withheld MMF/MPA or MTX reporting that their symptoms improved (Supplemental Figure 12). Flares at 4 weeks occurred in 5 (20%) of participants with SLE, all of which were considered mild/moderate, and 2 (6%) of participants with RA (Supplemental Table 8). For other disease types, severe flares were rare, occurring in only 1 participant with pemphigus (MMF/MPA withheld) (Supplemental Table 8). Importantly, none of the reported flares across any disease were deemed clinically meaningful, as they did not necessitate changes in medication.

## Discussion

In this randomized, multisite, open-label clinical trial, we found that a third mRNA vaccine enhances humoral responses to the SARS-CoV-2 RBD and spike proteins, regardless of whether IS medication was continued or withheld. Although lower than responses against the Wuhan-Hu-1 spike, humoral responses were also increased for other SARS-CoV-2 variants, including Omicron. Breakthrough infections occurred, but they were typically mild in all treatment groups. Additional vaccination was safe in individuals with ADs, with no vaccine-related SAEs or changes in global disease activity. Severe flares were rare, and none necessitated changes in treatment. Despite a limited sample size, we found similar trends in the humoral response in participants treated with the AD26.COV2.S vaccine.

We found that a third mRNA vaccine increases humoral responses to SARS-CoV-2 in individuals with ADs taking MTX, with no statistically significant difference in concentrations between those who withheld MTX and those who did not. However, numerical trends were observed, with a 3-fold increase in anti-RBD concentrations in individuals who withheld MTX. This finding is similar to the VROOM clinical trial, which found a 2-fold increase in anti-RBD titers in individuals who suspended MTX treatment for 2 weeks following a third mRNA or Oxford-AstraZeneca ChAdOx1 nCoV-19 (AZD1222) vaccine compared with those who did not (15). As a third vaccination with AZD1222 appeared to have a greater effect on the antibody response compared with BNT162b2 and mRNA-1273 in the VROOM clinical trial (15), we may not have seen a significant improvement in the antibody response, as we only analyzed mRNA vaccines. Supporting this hypothesis, the RESCUE 2 trial demonstrated a trend toward lower antibody levels in individuals with immune-mediated inflammatory disease who continued conventional synthetic disease-modifying antirheumatic drug regimens, the majority of which contained MTX, compared with those who withheld their medication or compared with healthy controls following a third vaccination with AZD1222; however, withholding these drugs did not impact the humoral response in individuals receiving a third mRNA vaccine (18). Also consistent with our findings, the VROOM clinical trial observed mild SARS-CoV-2 infections with similar frequencies throughout the study (15).

Results from observational studies are conflicting on the effects of MMF/MPA withholding around the time of initial and additional vaccination, and clinical trials are lacking in individuals with ADs (16, 19). A clinical trial in kidney transplant recipients on MMF/MPA found that a third or fourth dose of the mRNA vaccine increased antibody concentrations, with no difference in those who continued or withheld MMF/MPA for 1 week before and 1 week after the additional vaccine dose (20). In addition, neutralizing activity and SARS-CoV-2–specific T cell responses were similar in both groups (20, 21). While ongoing efforts, such as an NIH-funded trial (ClinicalTrials.gov NCT05077254), aim to explore the impact of reducing immunosuppression on vaccine responses in solid organ transplant patients, enrollment challenges have arisen due to evolving vaccine guidelines. To our knowledge, our study is the first randomized clinical trial of MMF/MPA withdrawal in individuals with ADs.

Although anti-RBD seropositivity increased from 6% to 36% 4 weeks after the third mRNA vaccination in BCDT-treated participants, it remained lower compared with MMF/MPA- and MTX-treated participants. This finding aligns with a European clinical trial in which only 32% of 28 individuals treated with rituximab seroconverted following a third mRNA vaccine (22). The reduced seropositivity following SARS-CoV-2 mRNA vaccination contrasts with responses to recall antigens, such as tetanus, where seropositivity rates remain unaffected by BCDT (23). This discrepancy likely arises because CD20, which is targeted by BCDT, is expressed on naive and memory B cells, which are critical for generating de novo antibody responses to novel antigens like SARS-CoV-2, but not on long-lived plasma cells that maintain preexisting antibodies against recall antigens such as tetanus (24). As previously described (25, 26), baseline CD19^+^ cell counts were associated with subsequent seropositivity against RBD, suggesting that waiting until B cells have repopulated before vaccination could improve vaccine responses in these individuals. Despite the lower antibody responses, breakthrough infection rates were similar across the treatment cohorts and those reported in the general population during the Delta and Omicron waves of COVID-19 (27–30). Importantly, most breakthrough infections were mild and none were fatal. Robust SARS-CoV-2–specific CD4^+^ and CD8^+^ T cell responses are observed in individuals treated with BCDT, similar to healthy controls, especially among those who did not develop anti-RBD antibodies (25). As SARS-CoV-2–specific T cells are associated with better COVID-19 outcomes (31, 32), T cell responses may provide an alternative protective mechanism in these individuals, compensating for lower humoral responses.

Consistent with observations in the general population (17), humoral responses to and neutralization of the Omicron variant were significantly lower compared with earlier SARS-CoV-2 strains. However, although our study occurred during the Omicron outbreak, subsequent infections were mild, with only 3 (2% of participants) nonfatal hospitalizations, reflecting a hospitalization risk comparable to that seen in the general population (33). These findings suggest that additional vaccination prevents severe outcomes against newer SARS-CoV-2 variants, even with reduced humoral responses. Since T cells are less impacted by spike protein mutations and continue to recognize mutant SARS-CoV-2 variants (34, 35), they may play a compensatory role in protecting against severe disease. Nevertheless, additional or variant-adapted vaccine doses would likely enhance immune protection, especially in individuals treated with BCDT, who generally exhibit lower humoral responses.

We observed minimal AEs and no significant impact on global disease activity following additional vaccination, reinforcing the safety of additional doses in individuals with ADs on IS therapies (36–38). Disease-specific flares occurred in 20% of patients with SLE, although all were mild/moderate, and 6% of participants with RA, consistent with reported flare rates after COVID-19 vaccination in individuals with ADs, which range from 0.4%–20%, depending on study design and population characteristics (39). A meta-analysis estimated a rate of 8% for mRNA vaccination, a higher rate compared with vector-based (0.32%) and inactive vaccines (3.07; *P* = 0.0086), although heterogeneity across studies was high (39). The rate of disease flare after COVID-19 vaccination may be higher compared with other vaccines. For example, one study found that patient-reported disease flares after COVID-19 vaccination were significantly higher than those after influenza vaccination (16% vs. 1%, respectively) (40). Importantly, most flares observed in our study and others (39) were mild to moderate and did not require treatment escalation, suggesting that while COVID-19 vaccines may pose a slightly higher risk of disease flares compared with other vaccines, their benefits outweigh these risks.

Notably, disease flares were minimal 4 weeks after the third mRNA vaccination in individuals who temporarily withheld MMF/MPA or MTX around the time of vaccination, with only 1 severe flare occurring among the 11 participants who withheld these medications. In the VROOM clinical trial, self-reported flares were initially higher in participants who withheld MTX compared with those who continued MTX 4 and 12 weeks after the third vaccination; however, these differences resolved within 26 weeks, with most flares not requiring medical attention (14, 15). Further supporting AD stability, autoantibody levels and type I IFN signature gene expression remained stable after both the second and third mRNA vaccine doses (38, 41). Together, these findings support the safety of additional mRNA vaccination in individuals with ADs who are on IS therapies, even among those who have withheld medications.

This study has some limitations. Due to recruitment challenges and updated vaccination guidance, we were unable to reach the target sample size, which limited our ability to analyze the effects of the mRNA vaccines separately or the AD26.COV2.S vaccine. However, we found that vaccine type was not significantly associated with treatment response among participants who received the mRNA vaccine. At later time points, many participants with suboptimal responses transitioned into Stage 2 of this study and were no longer followed, reducing the sample size for longitudinal analyses. Additionally, some of the participants treated with BCDT also received MTX or MMF/MPA, which could affect results. However, we found that withholding MMF/MPA or MTX did not significantly affect the humoral response to vaccination, suggesting that the primary IS effect in the BCDT group was due to BCDT itself rather than the other treatments. The trial was not powered to detect differences between the withhold and continue groups, nor for secondary or exploratory outcomes; further investigation in studies powered for these endpoints is needed. Furthermore, the evolving availability of outpatient antiviral therapies (e.g., Paxlovid, remdesivir) during the study period introduced variability in COVID-19 management. Despite this limitation, clinical outcomes were favorable, with only 3 nonfatal hospitalizations observed. Our study focused primarily on humoral responses and did not assess cellular immunity or Fc effector functions, which can protect against COVID-19, particularly for variants that escape neutralizing antibodies (42, 43). Finally, this study focused solely on the third mRNA and second AD26.COV2.S vaccine. As the COVID-19 vaccination strategies evolved to include additional boosters, our findings may not fully reflect current vaccination strategies. Future studies examining the effects of multiple boosters in this population would provide valuable insights into long-term vaccination strategies for individuals with ADs.

In conclusion, additional COVID-19 vaccination is both effective and safe for individuals with ADs treated with IS therapies. While we observed a numerical trend toward an increased humoral response in patients who withheld MMF/MPA or MTX around the time of vaccination, the difference did not reach statistical significance, and no safety concerns were identified. Our study, one of the few randomized clinical trials examining COVID-19 vaccination strategies in this underrepresented yet high-risk population, provides important evidence to guide vaccination approaches. These findings do not support a clear benefit from withholding these medications around vaccination; thus, current data support continued use of COVID-19 vaccines in this population without the need for adjusting IS treatments. However, further studies are warranted to explore strategies to improve humoral responses in individuals who maintain suboptimal responses, including the potential benefits of additional vaccine and/or heterologous doses and variant-specific vaccines.

## Methods

### Trial design and sex as a biological variable.

The COVID-19 Booster Vaccine in Autoimmune Disease Non-Responders (ClinicalTrials.gov NCT05000216) study was a randomized, multisite, adaptive, open-label clinical trial that included male and female participants. This study consisted of 2 stages: Stage 1 participants received an additional homologous vaccine dose, and Stage 2 participants received an additional heterologous vaccine dose; only Stage 1 results are reported here. Additional vaccination for Stage 1 is defined as a third dose of BNT162b2 (Pfizer-BioNTech) or mRNA-1273 (Moderna) or a second dose of AD26.COV2.S (Janssen). Outcomes in male and female participants are reported together. Study vaccines were provided by Health and Human Services Coordination Operations and Response Element, formerly Countermeasures Acceleration Group and Operation Warp Speed, and distributed to each clinical site by the Division of Allergy, Immunology, and Transplantation/NIAID’s Clinical Products Center. The full trial protocol is provided in the supplemental material. Patients and members of the public were not involved in the design, conduct, or reporting of this trial.

### Eligibility criteria.

Eligible participants were aged 18 years and older and met at least one set of 2019 American College of Rheumatology (ACR)/European Alliance of Associations for Rheumatology (EULAR) (44) or 2012 SLICC classification criteria (45) for SLE, 2010 ACR/EULAR classification criteria for RA (46), 2013 ACR/EULAR classification criteria for SSc (47), 2017 McDonald criteria for MS (48), or the international consensus criteria for pemphigus (49). Participants were also required to currently be taking MMF (minimum of 1000 mg/day for at least 8 weeks) or MPA (minimum of 720 mg/day for at least 8 weeks), MTX (minimum of 7.5 mg/week for at least 8 weeks), or an anti-CD20 or anti-CD19 BCDT in the previous 18 months. Participants had to have received 2 primary doses of the BNT162b2 (Pfizer-BioNTech) or mRNA-1273 (Moderna) vaccine or 1 dose of the AD26.COV2.S (Janssen) vaccine at least 4 weeks and no more than 52 weeks prior to screening and have had a negative (<0.79 U/mL, Roche Elecsys Anti-SARS-CoV-2 S) or suboptimal (≤200 U/mL, Roche Elecsys Anti-SARS-CoV-2 S) response to the initial COVID-19 vaccine regimen. Exclusion criteria included history of severe allergic reaction to the initial COVID-19 vaccine regimen or any component of the COVID-19 vaccine, female participants planning a pregnancy during the trial, receipt of a COVID-19 booster vaccine, receipt of anti–SARS-CoV-2 monoclonal antibodies or plasma products within 90 days of screening, history of thrombosis in the previous 12 months, history of SARS-CoV-2 infection within 30 days of screening, concurrent treatment with cyclophosphamide, cladribine, alemtuzumab, mitoxantrone, or other investigational agents, and ongoing dialysis or history of a solid organ transplant. Participants with active disease resulting in an inability to withhold IS therapy or necessitating increased doses or the addition of new IS medication in the MTX or MMF/MPA arms were also excluded. The maximum corticosteroid dose allowed was a prednisone equivalent of 10 mg daily.

### Sampling and randomization of participants.

At the screening visit, inclusion and exclusion criteria were assessed, including Roche Elecsys Anti-SARS-CoV-2 S assays to evaluate the initial vaccine response and autoimmune disease activity (Supplemental Figure 1). Participants were assigned to cohorts defined by their IS medication (MMF/MPA-, MTX-, or BCDT-treated) and their original COVID-19 vaccine regimen. If a participant had received BCDT within the previous 18 months and was also on MMF/MPA or MTX, they were included in the BCDT cohort. Participants in the MMF/MPA- and MTX-treated cohorts were randomized one-to-one within the cohorts to continue or withhold their IS medication. Study site staff randomized participants through a centralized, web-based, password-protected randomization system. Randomization was performed using a permuted block design stratified by disease type.

### Trial procedure.

All participants received an additional (third dose of BNT162b2 or mRNA-1273 and second dose of AD26.COV2.S) homologous dose of COVID-19 vaccine at the baseline visit, within 4 weeks of the screening visit. In the withhold groups, MMF/MPA was withheld for 3 days before and 10 days after the additional vaccine, while MTX was withheld for at least 7 days before and at least 7 days after the additional vaccine, but for no more than 21 days total. Participants randomized to continue MMF/MPA or MTX were required to maintain a stable dose during the vaccination period. Participants in the BCDT cohort continued to take BCDT without prespecified changes in schedule and dosing. All participants were asked to continue taking stable doses of any additional IS medication unless changes were required due to disease activity.

### Antibody responses.

Antibody responses to Wuhan-Hu-1 full-length spike, RBD, and N proteins were measured using the V-PLEX SARS-CoV-2 384 Panel 1 IgG kit (catalog K25392U, Meso Scale Diagnostics) as previously described (50) at baseline and 4, 12, 24, 36, and 48 weeks after vaccination. Anti-spike, -RBD, and -N seropositivity was defined as concentrations above the positive threshold value determined during assay validation with pre-2019 samples (spike 10.842 IU/mL; RBD 14.086 IU/mL) (50). Antibody responses to spike proteins of different SARS-CoV-2 variants (Wuhan-Hu-1, Alpha [B.1.1.7], Beta [B.1.351], Delta [B.1.617.2], and Omicron [BA.1, BA.2, BA.2.12.1, BA.2.75, BA.5]) were measured as AU/mL relative to a reference standard using the V-PLEX SARS-CoV-2 Key Variant Spike Panel 1 IgG kit (catalog K15651U, Meso Scale Diagnostics), as previously described (51).

### Neutralization.

Plasma (1:40 initial dilution) was serially diluted 1:2 across a 96-well plate and mixed with enough virus (isolate USA-WA1/2020) to yield a final multiplicity of infection of 0.01. After incubating the antibody virus mixture for 1 hour at 37°C, the virus mixture was transferred to a 96-well plate containing VERO E6 cells seeded at 10,000 cells per well. SARS-CoV-2 activity was determined 96 hours after infection by visually observing cytopathic effects. The antibody dilution at which virus-positive wells were observed was recorded.

The V-PLEX ACE2 Neutralization Kit (catalog K15654U, Meso Scale Diagnostics) was used to detect surrogate neutralizing antibodies across SARS-CoV-2 variants (Wuhan-Hu-1, Alpha [B.1.1.7], Beta [B.1.351], Delta [B.1.617.2], and Omicron [BA.1, BA.2, BA.2.12.1, BA.2.75, BA.5), as previously described (51). Neutralization was quantified in serum by reduction in electrochemiluminescence signal, with results reported as fold inhibition relative to diluent-only controls (representing maximum ACE2 binding to the antigen), reflecting competitive neutralization activity.

### Outcomes.

The primary outcome was the increase in anti-RBD antibody concentrations from baseline to 4 weeks after additional homologous COVID-19 vaccination. Secondary outcomes related to antibody response included change in anti-RBD seropositivity from baseline to 4 weeks after additional COVID-19 vaccination, associations between anti-RBD concentrations and clinical parameters, seropositivity and/or antibody concentrations for other SARS-CoV-2 antigens and variants, neutralization, and longitudinal changes in antibody responses. Exploratory analyses assessed whether baseline CD19^+^ B cell counts and timing since last BCDT dose were associated with post–additional vaccination anti-RBD responses in the BCDT-treated cohort. Additional secondary outcomes included changes in global disease activity, as measured by the Clinical Global Impression of Change (CGI-C) (52) and the Physician Global Assessment (PGA) (53), and changes in patient-reported outcomes, as measured by the Patient Global Assessment (PtGA) (53) and Patient Global Impression of Change (PGI-C) (54) across all 5 diseases. Disease-specific flares were also measured using the following disease-specific assessments: Thanou-modified SELENA-SLEDAI Flare Index (55) for SLE participants, an increase in the Disease Activity Score-28 CRP (56) score of greater than 0.2 or greater than 0.6 if the week 4 score was greater than 3.2 for RA, onset of new or significant worsening of internal organ involvement requiring hospitalization or change in treatment or worsening of skin thickening on the modified Rodnan Skin Score (mRSS) (57) greater than 4 units for SSc, physician-assessed relapse for MS, and at least 3 new lesions a month that do not heal spontaneously within 1 week or by the extension of established lesions in a participant who has achieved disease control for pemphigus.

Safety outcomes included the proportion of participants who experienced any Grade 1 or higher AE related to the COVID-19 vaccine, the incidence of solicited AEs that were local and systemic events of reactogenicity, and any SAE, MAAE, NOCMC, or COVID-19 diagnosis. Adverse events of special interest included the incidence of myocarditis and pericarditis. Solicited events of reactogenicity were reported from day 1 through day 7, AEs were reported from day 1 through day 28, and SAEs, MAAEs, NOCMCs, and COVID-19 were reported from day 1 through the end of study participation. AEs were graded according to the Common Terminology Criteria for Adverse Events, version 5.0 (58). COVID-19 diagnosis was confirmed by molecular COVID-19 testing. Visits to assess endpoints occurred at baseline (week 0) and weeks 4, 12, 24, 36, and 48.

### Statistics.

Stage 1 of this trial was designed to enroll up to 900 adult participants across 15 arms, defined by IS treatment (MMF/MPA, MTX, or BCDT), vaccine type (mRNA-1273, BNT162b2, or Ad26.COV2.S), and randomization to withhold or continue IS treatment (MMF/MPA and MTX cohorts only). In each treatment arm, up to 60 participants were to be randomized or allocated. The number of participants with results ≤50 U/mL and with results >50 U/mL and ≤200 U/mL was capped at 40 participants per arm. Sample sizes were defined to test whether responses to the additional vaccine dose were greater than an ineffective rate of seroprotection that was less than or equal to 0.25 (null hypothesis). Under this assumption, a sample size of 33 evaluable participants per arm achieved at least 90% power to reject the null hypothesis when the true response rate is 0.5 or greater, using a 1-sided exact test with α = 0.05. A sample size of 40 participants per arm was originally planned to account for a 20% dropout rate. Later versions of the protocol amended the sample size to 60 participants per arm to account for expansions in the Roche Elecsys eligibility criteria. Due to recruitment challenges, actual enrollment per arm was lower than planned.

A validated threshold for protective antibody responses has not been established for the Meso Scale Diagnostics SARS-CoV-2 assays, so vaccine response is evaluated using seropositivity rates (defined as having a result greater than the positive threshold value) and a continuous measure of antibody concentrations. Descriptive statistics included median, minimum, maximum, interquartile range, geometric mean, coefficient of variation of the geometric mean, and 95% confidence intervals for the Wuhan-Hu-1 spike and RBD proteins. For concentrations below the lower limit of detection (LLOD), numeric values equivalent to LLOD/2 were assigned; concentrations above the upper limit of quantification were reported without adjustments. Antibody results collected after a documented COVID-19 diagnosis, monoclonal antibody use, or a COVID-19 vaccine given off-study were excluded from the analyses. Descriptive statistics were reported in the vaccinated population (all participants who received an additional vaccination) within each group (defined by cohort, vaccine, and IS treatment plan) and in the combined groups that received an mRNA vaccine.

Categorical variables were summarized with frequencies and proportions. The Wilcoxon signed-rank test assessed changes in antibody concentrations within arms and subgroups over time. Comparisons across arms and predictors of response were assessed using the Wilcoxon rank-sum test and stratified van Elteren’s tests. Changes in the seropositivity rate over time were evaluated using McNemar’s test. The correlation between immune response endpoints is explored in the vaccinated population using Spearman’s rank correlation coefficients. The cumulative incidence of breakthrough COVID-19 was calculated using the Kaplan-Meier product limit estimator using Greenwood’s formula for standard error. Statistical analyses were performed using SAS 9.3 or higher (SAS Institute). *P* values were not adjusted for multiple comparisons. *P* values of less than 0.05 were considered statistically significant.

### Study approval.

Participants were recruited from 19 US centers (Supplemental Table 9), and all provided written informed consent before participation at the screening visit. The study was approved by a central institutional review board (Advarra, Columbia, Maryland, USA), reviewed by institutional review boards at each site, and conducted following the Declaration of Helsinki. An independent Data and Safety Monitoring Board reviewed the progress of the study and safety data twice per year.

### Data availability.

All data are accessible from ImmPort (www.immport.org) under study accession SDY3324.

## Author contributions

MM, AP, WTB, EG, JM, ABO, DK, and JAJ designed the research and KS, JMG, SM, JW, GD, MC, RC, SN, BCL, LS, and AM conducted experiments. MM, AP, JAC, ZSW, AK, JAS, SL, AS, JW, GD, MC, RC, AHJK, DLK, FK, ADA, KS, JMG, GP, YMD, SM, MAS, SDH, MV, SW, KY, ST, RC, SN, BCL, LS, AM, WTB, ASP, AB, DK, and JAJ acquired data and WTB, AP, CAW, MM, ABO, DK, and JAJ analyzed data. All authors wrote and/or reviewed the manuscript.

## Funding support

This work is the result of NIH funding, in whole or in part, and is subject to the NIH Public Access Policy. Through acceptance of this federal funding, the NIH has been given a right to make the work publicly available in PubMed Central.

- Autoimmunity Centers of Excellence, a research network funded by NIAID/NIH grants U19AI144306, U19AI082714, U19AI110483, UM1AI110494, UM1AI144292, UM1AI144295, UM1AI144288, UM1AI144298, and UM1AI110557.
- NIAID/NIH grants UM2AI117870, U19AI110483-08S4, and 75N93022C00003 (to Rho, the statistical and clinical coordinating center).

## Supplementary Material

Supplemental data

ICMJE disclosure forms

Supporting data values

## Figures and Tables

**Table 1:**
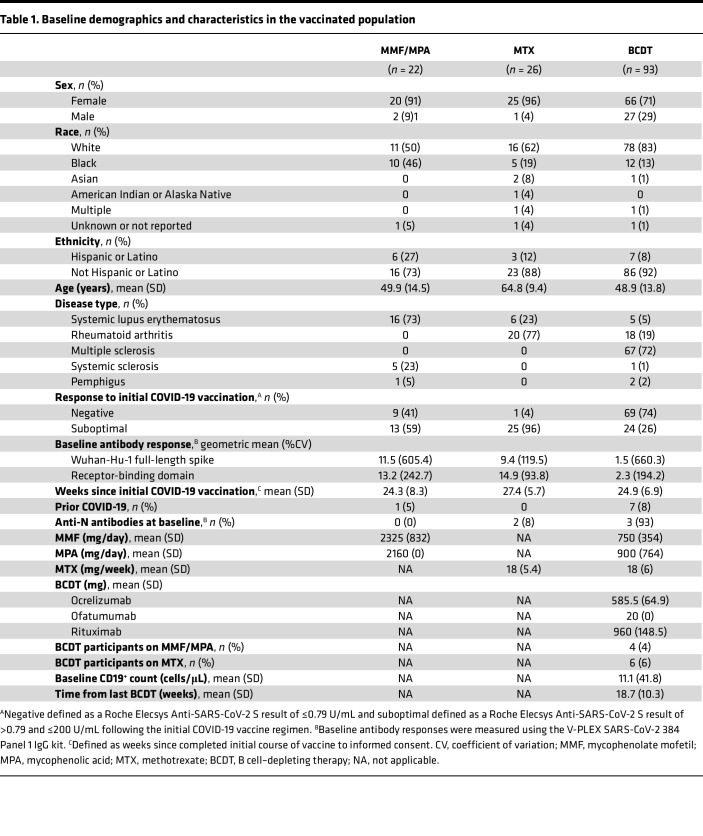
Baseline demographics and characteristics in the vaccinated population

**Table 2:**
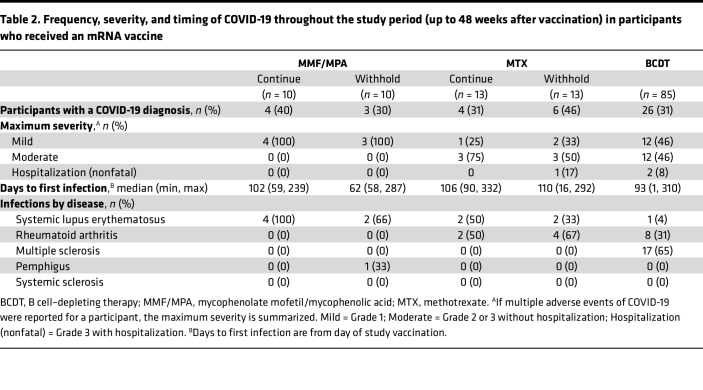
Frequency, severity, and timing of COVID-19 throughout the study period (up to 48 weeks after vaccination) in participants who received an mRNA vaccine

## Version 1 11/25/2025

In-Press Preview

## Version 2 01/09/2026

Electronic publication
