# Supplementary material for: Prospective SARS-CoV-2 additional vaccination in immunosuppressant-treated individuals with autoimmune diseases in a randomized controlled trial
Source: JCI Insight. 2025 Nov 25;11(1):e191266. doi: 10.1172/jci.insight.191266 (PMC12890511; doi:10.1172/jci.insight.191266)
Supplement: Supplemental data [file jciinsight-11-191266-s239.pdf]

191266

Conflict of interest:

JAC received personal compensation for consulting for Astoria, Atara, Biogen, Bristol-Myers Squibb, Convelo, and Viatris. ASP received equity, payments, and research funding from Cabaletta Bio and provided consulting to Janssen, Sanofi, and Avilar.

ASP reports patent royalties from US Patent PCT/US2015/028872, WO/2015/168613A3, US Patent 10,301,370 B2 ("Compositions and methods of chimeric autoantibody receptor T cells") and US Patent PCT/US2019/035409, WO/2019/236593A1 ("Compositions and methods of muscle specific kinase chimeric autoantibody receptor T cells"), both licensed by Calaletta Bio.

AHJK reports US Patent 11029318B2 ("Methods for predicting and treating patients with increased risk of adverse pregnancy outcome") with Kypha.

JAS has received research support from Boehringer Ingelheim, Bristol Myers Squibb, Johnson & Johnson, and Sonoma Biotherapeutics unrelated to this work. He has performed consultancy for AbbVie, Amgen, Boehringer Ingelheim, Bristol Myers Squibb, Fresenius Kabi, Gilead, Inova Diagnostics, Johnson & Johnson, Merck, MustangBio, Novartis, Optum, Pfizer, ReCor, Sana, Sobi, and UCB unrelated to this work.

YMD has served as a consultant and/or received grant support from Biogen Idec, Celgene/Bristol Myers Squibb, EMD Serono, Sanofi-Genzyme, Roche-Genentech, Novartis, Questor, Janssen, and Teva Neuroscience.

JAJ has received consulting funds from GSK and has IP with OMRF licensed to Progentec Biosciences. JAJ reports US Patent 20240011983 ("Method for determining whether a systemic lupus erythematosus (sle) patient is undergoing a pre-flare event") with OMRF licensed to Progentec Biosciences.

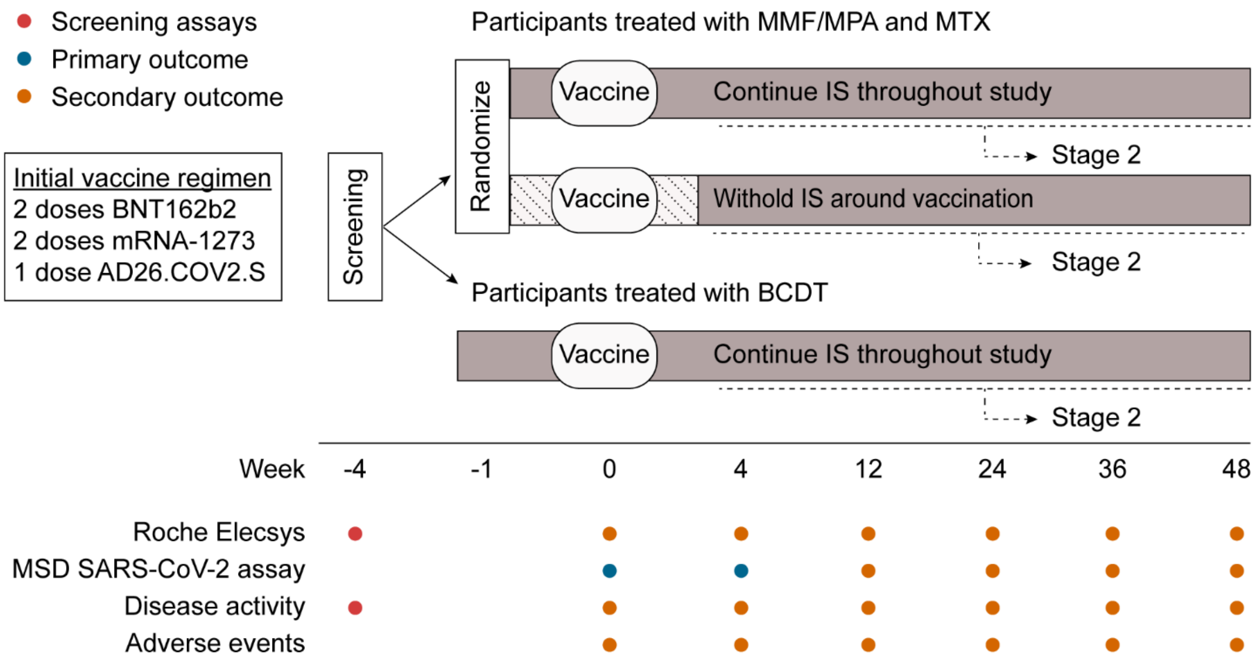

Supplemental Figure 1. Schematic overview of the study design. After screening (week -4), participants with autoimmune diseases treated with MMF/MPA, MTX, or BCDT who had completed an initial COVID-19 vaccine regimen (2 doses BNT162b2, 2 doses mRNA-1273, or 1 dose AD26.COV2.S) and met all eligibility criteria were enrolled. All participants received an additional COVID-19 vaccine dose at week 0. Participants on MMF/MPA or MTX were randomized to either continue or withhold immunosuppression (IS) around the time of additional vaccination; participants on BCDT continued IS throughout. Assessments included screening assays (Roche Elecsys assay; red dots), primary outcome (MSD SARS-CoV-2 assay; blue dots), and secondary outcomes (disease activity, adverse events; orange dots) at the indicated time points. Participants with suboptimal humoral responses after Stage 1 were eligible to proceed to Stage 2 (not reported here). Roche Elecsys and MSD SARS-CoV-2 assays, disease activity, and adverse events were evaluated as shown in the assessment schedule.

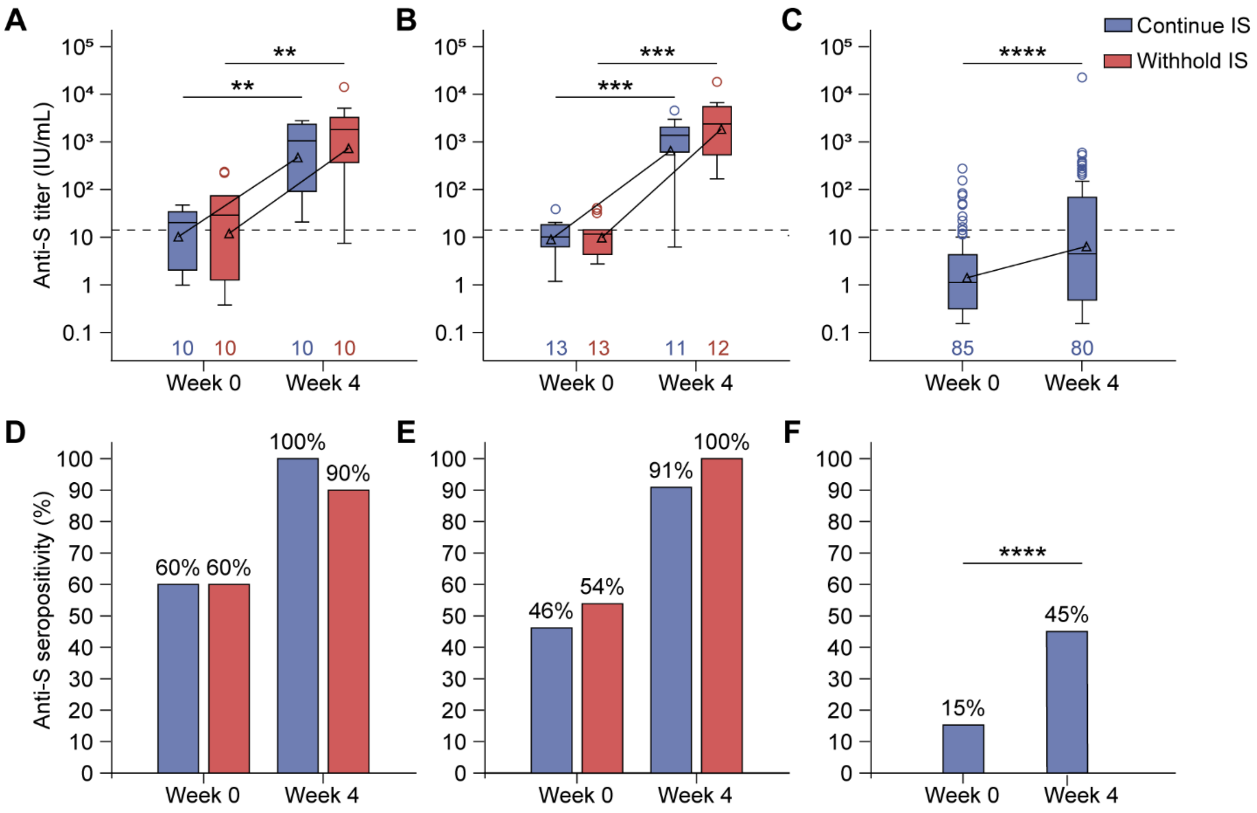

Supplemental Figure 2. Anti-Spike (S) concentrations and seropositivity in participants treated with mycophenolate mofetil/mycophenolic acid (MMF/MPA)-, methotrexate (MTX)-, and B cell-depleting therapy (BCDT) who received a third mRNA vaccine. Concentrations of anti-spike antibodies at baseline and 4 weeks post-third vaccination in participants in the (**A**) MMF/MPA-, (**B**) MTX-, or (**C**) BCDT cohorts. Box plots display the interquartile range (IQR), with the line within the box indicating median values. Geometric mean values are represented by triangles and connected across study visits. Whiskers extend to 1.5*IQR, with circles indicating outliers. The dashed line represents the positivity cut-off, and numbers indicate the number of participants analyzed at each time point. The Wilcoxon signed-rank test was used to assess the change in antibody concentration from baseline to Week 4 within each group. No significant difference in concentrations in those who continued or withheld immunosuppressants using a van Elteren test. Seropositivity of anti-S antibodies at baseline and 4 weeks post-third vaccination in participants in the (**D**) MMF/MPA-, (**E**) MTX-, or (**F**) BCDT cohorts. Statistical significance was determined using a McNemar test. **p<0.01; ***<0.001; ****p<0.0001.

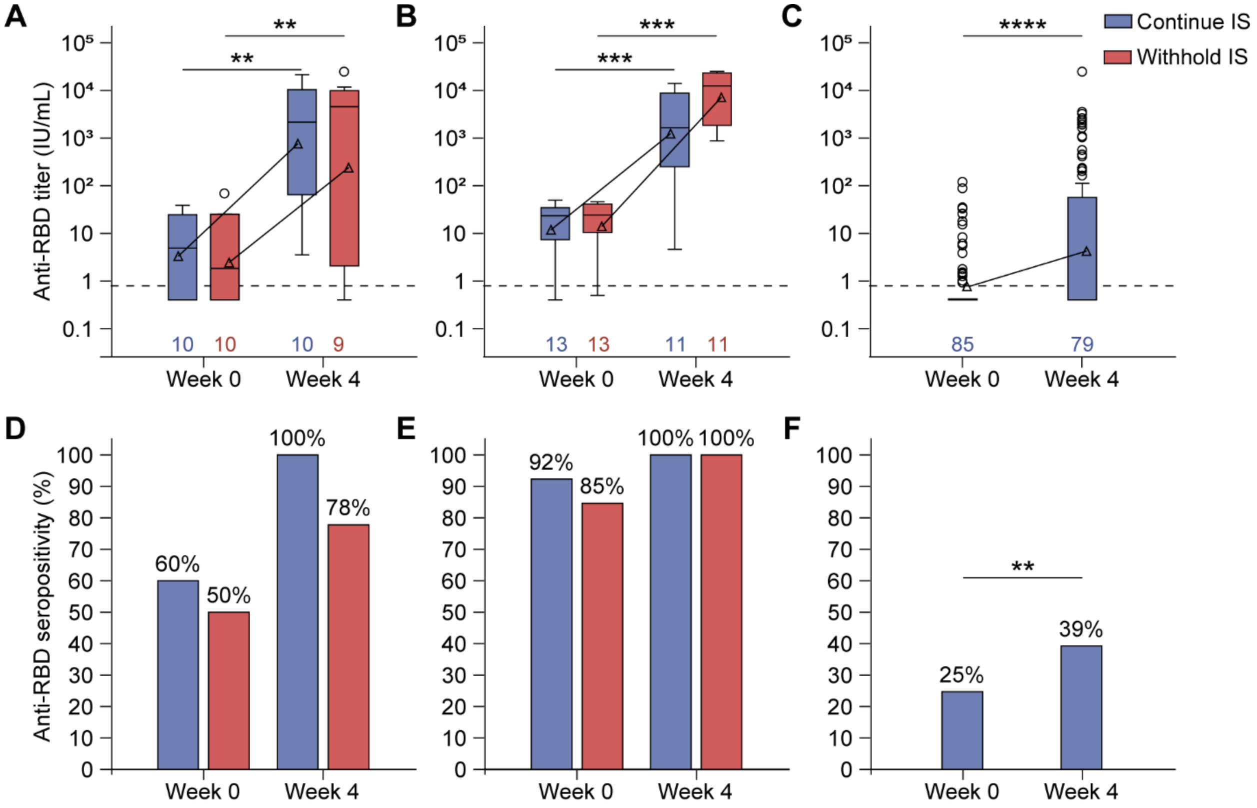

Supplemental Figure 3. Roche Elecsys® anti-spike concentrations and seropositivity in participants treated with mycophenolate mofetil/mycophenolic acid (MMF/MPA)-, methotrexate (MTX)-, and B cell-depleting therapy (BCDT) who received a third mRNA vaccine. Concentrations of anti-RBD antibodies at baseline and 4 weeks post-third vaccination in participants in the (**A**) MMF/MPA-, (**B**) MTX-, or (**C**) BCDT cohorts. Box plots display the interquartile range (IQR), with the line within the box indicating median values. Geometric mean values are represented by triangles and connected across study visits. Whiskers extend to 1.5*IQR, with circles indicating outliers. The dashed line represents the positivity cut-off, and numbers indicate the number of participants analyzed at each time point. The Wilcoxon signed-rank test was used to assess the change in antibody concentration from baseline to Week 4 within each group. Seropositivity of Roche Elecsys® anti-RBD antibodies at baseline and 4 weeks post-third vaccination in participants in the (**D**) MMF/MPA-, (**E**) MTX-, or (**F**) BCDT cohorts. Statistical significance was determined using a McNemar test. No significant difference in concentrations in those who continued or withheld immunosuppressants using a van Elteren test. **p<0.01; ***<0.001; ****p<0.0001.

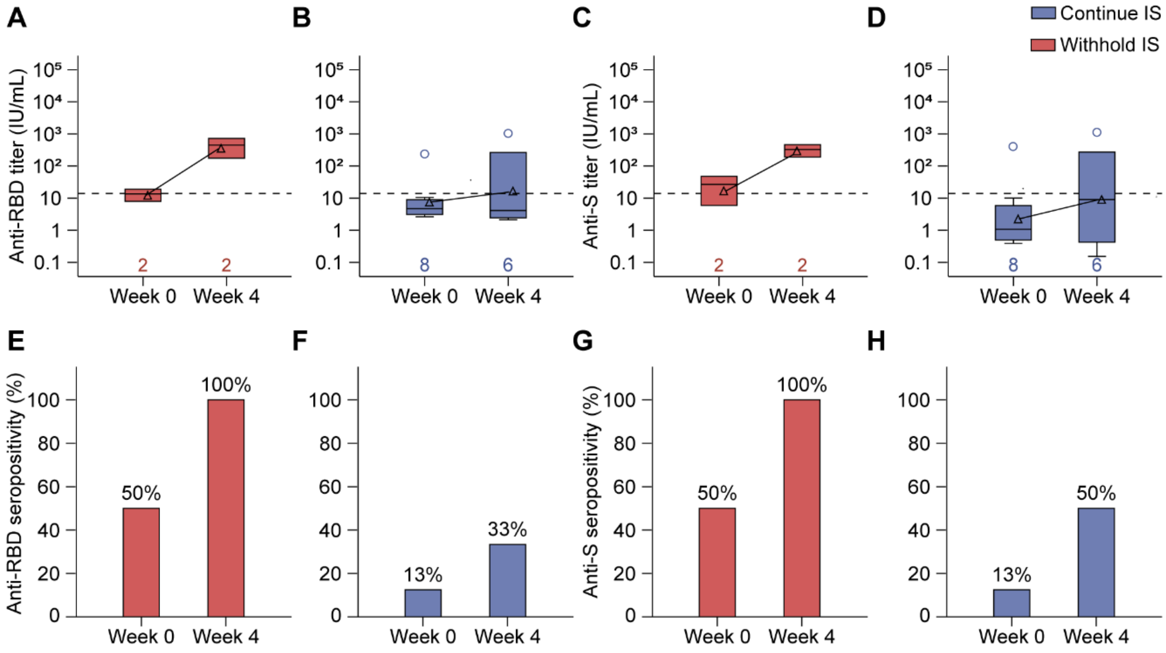

Supplemental Figure 4. Humoral response in participants who received a second AD26.COV2.S vaccine. (**A-D**) Concentration and (**E-F**) seropositivity of (**A-B** and **E-F**) anti-RBD and (**C-D** and **G-H**) anti-Spike (S) antibodies at weeks 0 and 4 post-second AD26.COV2.S vaccination in participants who withdrew (**A, C, E,** and **G**) mycophenolate mofetil/mycophenolic acid (MMF/MPA) or (**B, D, F,** and **H**) those taking B cell-depleting therapy (BCDT). Box plots display the interquartile range (IQR), with the line within the box indicating median values. Geometric mean values are represented by triangles and connected across study visits. Whiskers extend to 1.5*IQR, with circles indicating outliers. The dashed line represents the positivity cut-off, and numbers indicate the number of participants analyzed at each time point.

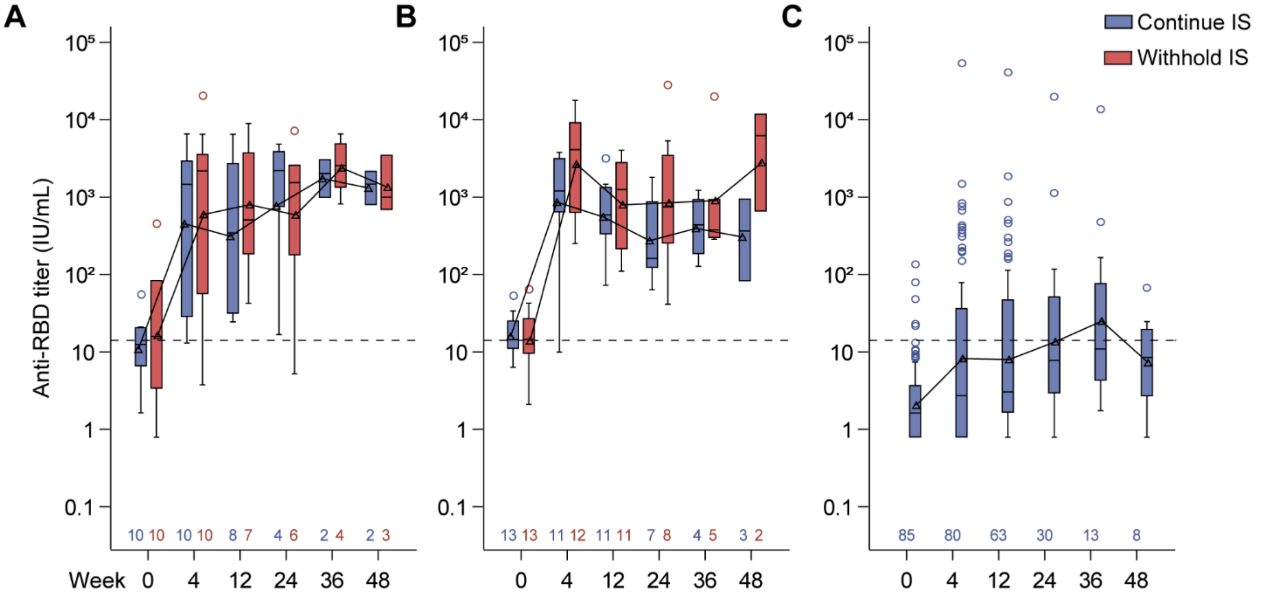

Supplemental Figure 5. Longitudinal anti-RBD responses post-third mRNA vaccination in participants treated with mycophenolate mofetil/mycophenolic acid (MMF/MPA)-, methotrexate (MTX)-, and B cell-depleting therapy (BCDT). Anti-RBD antibodies were measured at baseline and at 4, 12, 24, 36, and 48 weeks post-third vaccination in participants in the (A) MMF/MPA, (B) MTX, or (C) BCDT cohorts. Box plots display the interquartile range (IQR), with the line within the box indicating median values. Geometric mean values are represented by triangles and connected across study visits. Whiskers extend to 1.5*IQR, with circles indicating outliers. The dashed line represents the positivity cut-off, and numbers indicate the number of participants analyzed at each time point; samples collected after a documented COVID-19 diagnosis, monoclonal antibody use, or a COVID-19 vaccination given off-study are excluded from the analyses. Some participants with low or sub-optimal responses rolled over to Stage 2 of the study prior to completing Stage 1.

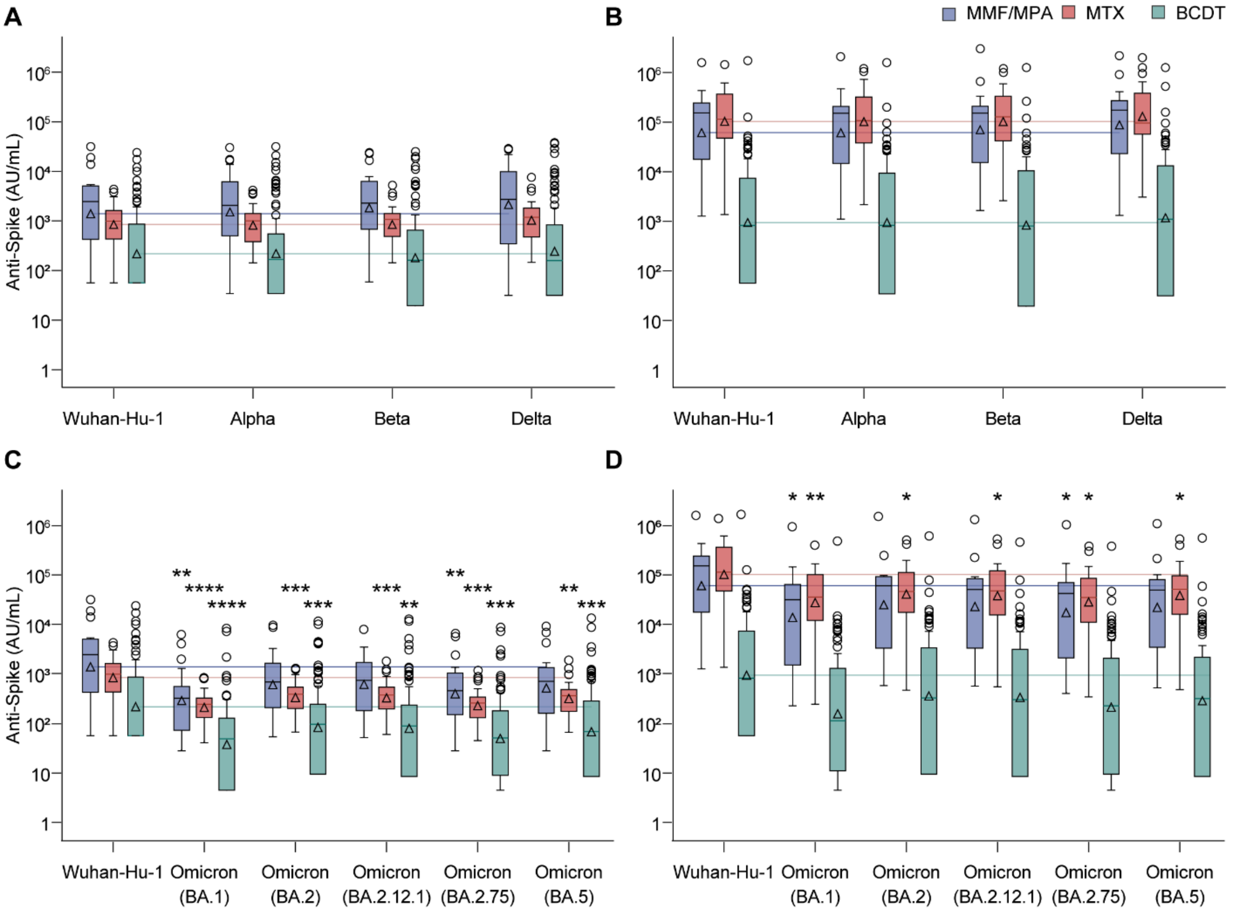

Supplemental Figure 6. Anti-Spike (S) humoral responses across SARS-CoV-2 variants. Antibody titers against the (**A-B**) Alpha, Beta, and Delta and (**C-D**) Omicron BA1, BA2, BA2.12.1, BA2.75, and BA5 S proteins relative to Wuhan-Hu-1 at (**A** and **C**) baseline and (**B** and **D**) week 4 post-third mRNA booster vaccination. Box plots display the interquartile range (IQR), with the line within the box indicating median values. Geometric mean values are represented by triangles. Whiskers extend to 1.5*IQR, with circles indicating outliers. Horizontal lines indicate the geometric mean for the Wuhan-Hu-1 Spike variant by cohort for each visit. Statistical significance was determined using the stratified van Elteren test. *$p<0.05$, **$p<0.01$ relative to the Wuhan-Hu-1 S protein for each cohort.

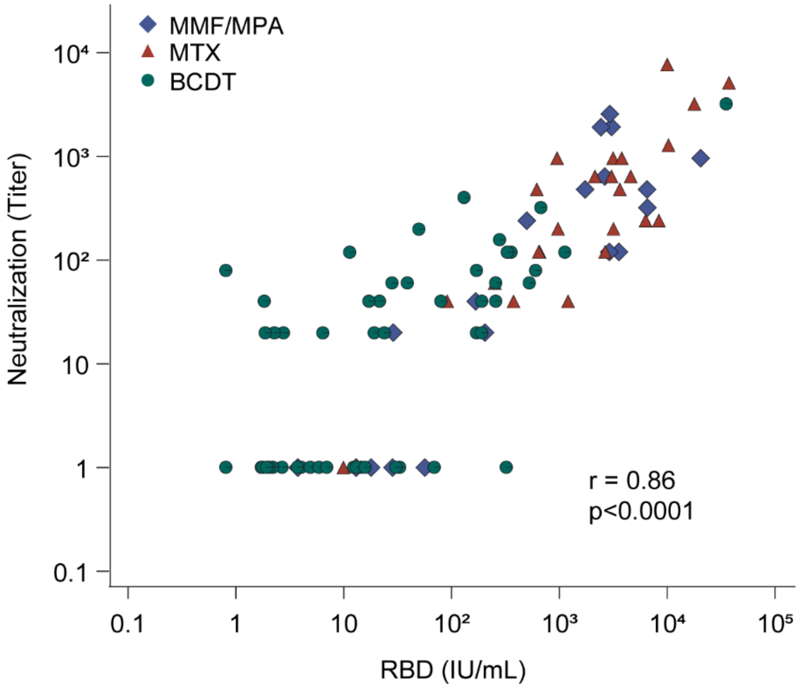

Supplemental Figure 7. Correlation of week 4 anti-RBD and neutralization titers in participants who received a third mRNA vaccine. Correlation between week 4 anti-RBD antibody titers and neutralization titers against the USA-WA1/2020 isolate. Correlation was assessed using Spearman's rank correlation coefficients.

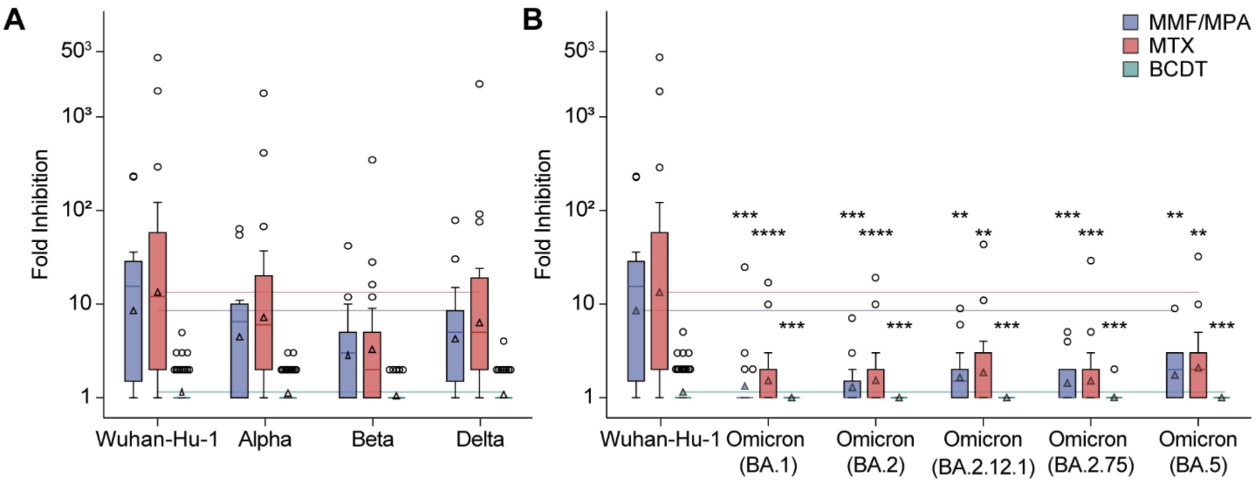

Supplemental Figure 8. ACE2 neutralization across SARS-CoV-2 variants. Fold inhibition of ACE2 binding against the (**A**) Alpha, Beta, and Delta and (**B**) Omicron BA.1, BA.2, BA.2.12.1, BA.2.75, and BA.5 SARS-CoV-2 variants relative to Wuhan-Hu-1 at week 4 post-third mRNA booster vaccination. Box plots display the interquartile range (IQR), with the line within the box indicating median values. Geometric mean values are represented by triangles and connected across study visits. Whiskers extend to 1.5*IQR, with circles indicating outliers. Horizontal reference lines indicate the geometric mean for the Wuhan-Hu-1 variant by cohort for each visit. Statistical significance was determined using the stratified van Elteren test. **p<0.01, ***p<0.001, ****p<0.0001 relative to Wuhan-Hu-1 for each cohort.

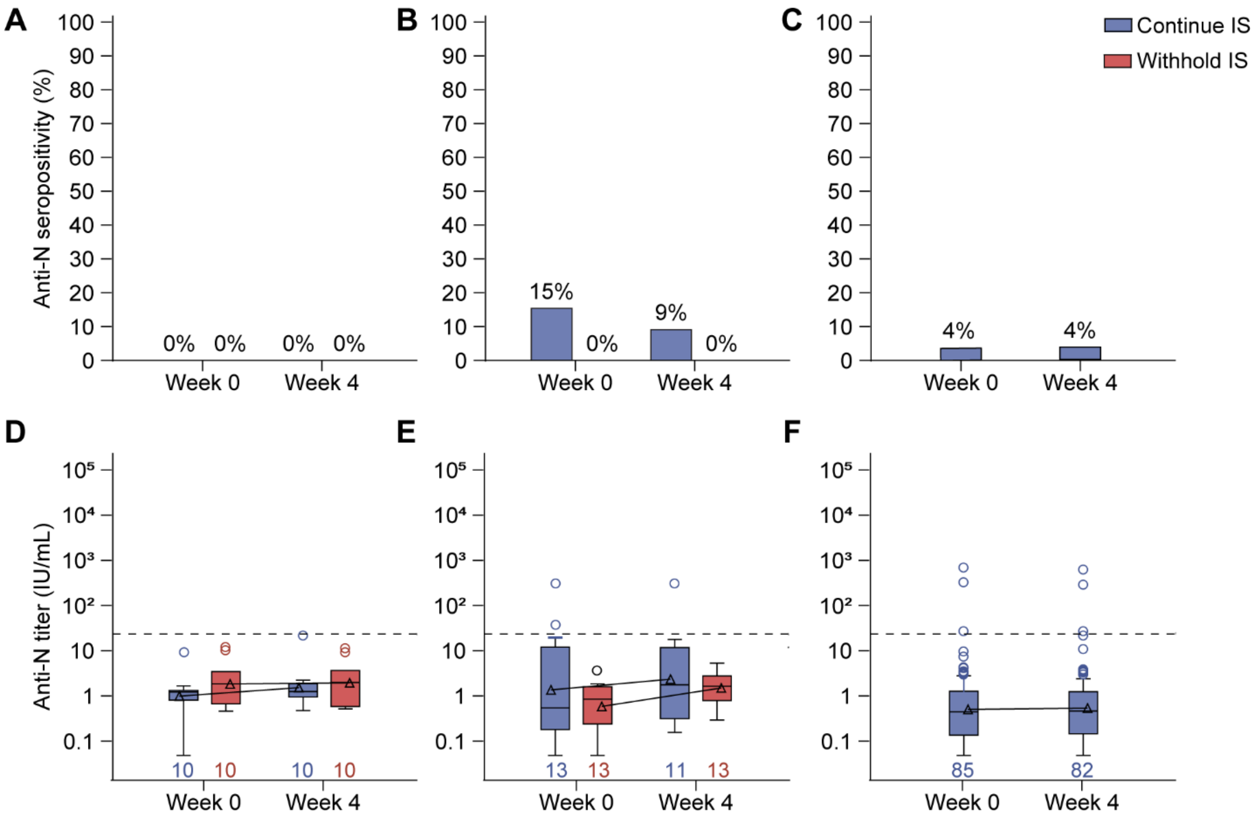

Supplemental Figure 9. Anti-N-protein antibodies in participants who received a third mRNA vaccine. Seropositivity of anti-N-protein antibodies at baseline and 4 weeks post-third mRNA vaccination in participants in the (**A**) mycophenolate mofetil/mycophenolic acid (MMF/MPA)-, (**B**) methotrexate (MTX)-, or (**C**) B cell-depleting therapy (BCDT) cohorts. Concentrations of anti-RBD antibodies at baseline and 4 weeks post-third mRNA vaccination in participants in the (**D**) MMF/MPA-, (**E**) MTX-, or (**F**) BCDT cohorts. Box plots display the interquartile range (IQR), with the line within the box indicating median values. Geometric mean values are represented by triangles and connected across study visits. Whiskers extend to 1.5*IQR, with circles indicating outliers. The dashed line represents the positivity cut-off, and numbers indicate the number of participants analyzed at each time point.

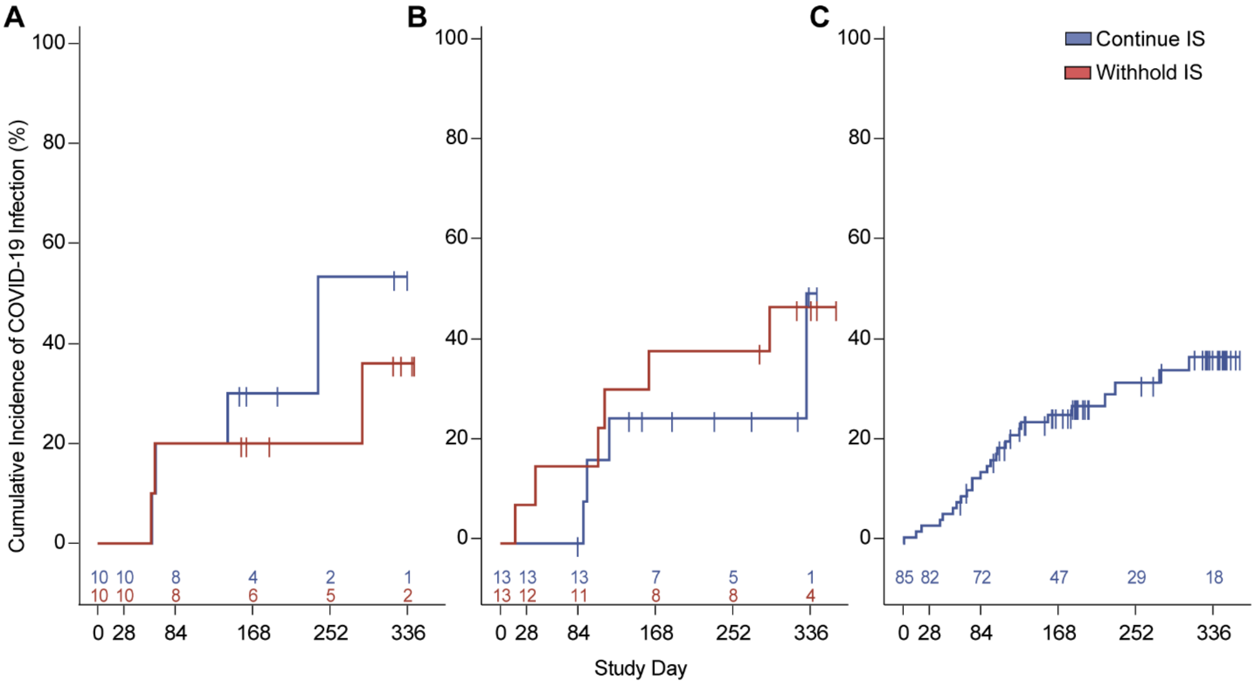

Supplemental Figure 10. Cumulative incidence of COVID-19 in the vaccinated population that received a third mRNA vaccine. Cumulative incidence of COVID-19 in participants within the (**A**) mycophenolate mofetil/mycophenolic acid (MMF/MPA)-, (**B**) methotrexate (MTX)-, or (**C**) B cell-depleting therapy (BCDT) cohorts throughout 48 weeks post-third vaccination. The cumulative incidence of COVID-19 was calculated using the Kaplan-Meier product limit estimator. Vertical lines indicate the timing of censored events.

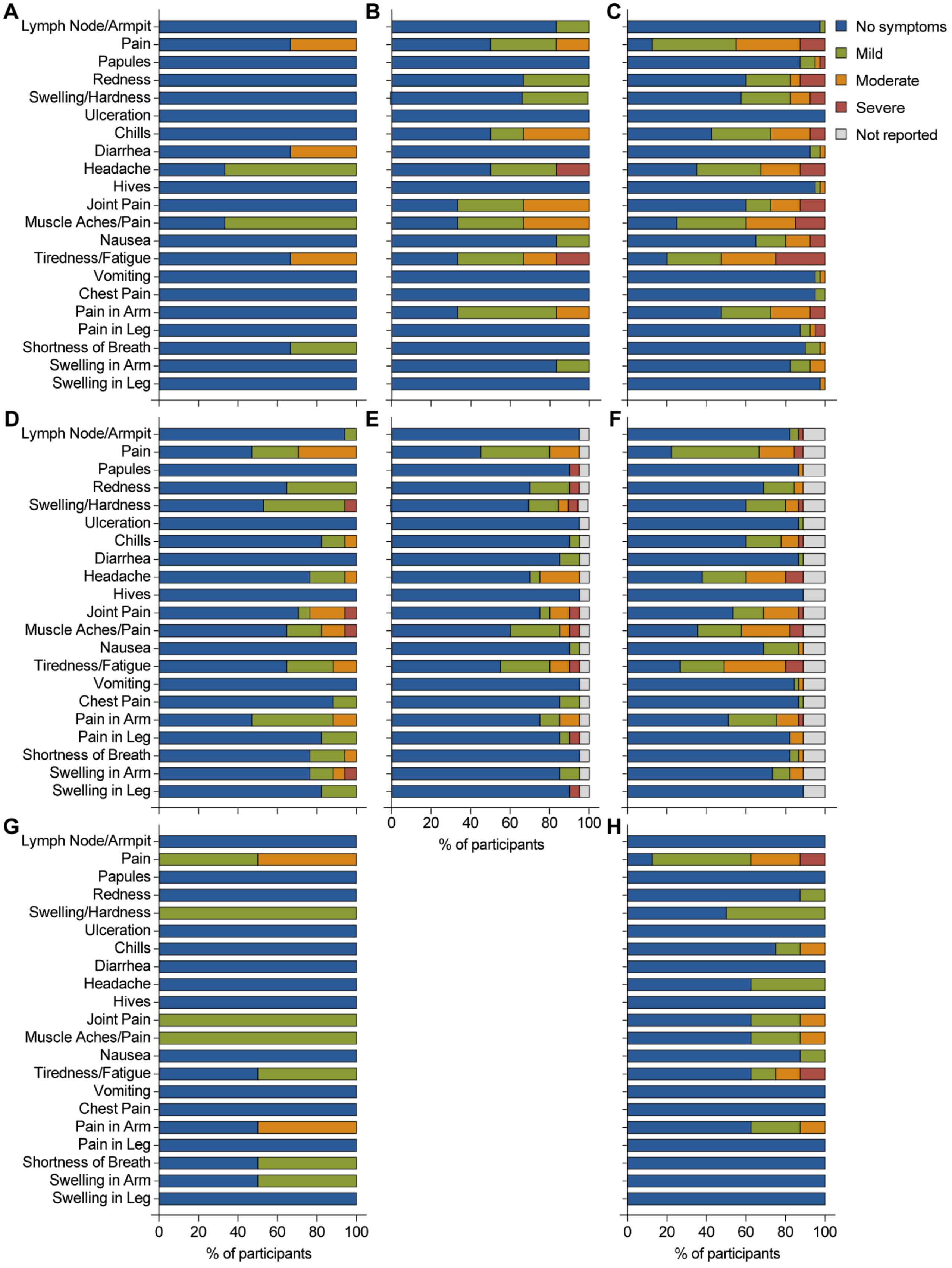
A
B
C
D
E
F
G
H
Lymph Node/Armpit
Pain
Papules
Redness
Swelling/Hardness
Ulceration
Chills
Diarrhea
Headache
Hives
Joint Pain
Muscle Aches/Pain
Nausea
Tiredness/Fatigue
Vomiting
Chest Pain
Pain in Arm
Pain in Leg
Shortness of Breath
Swelling in Arm
Swelling in Leg
No symptoms
Mild
Moderate
Severe
Not reported
0 20 40 60 80 100
% of participants

Supplemental Figure 11. Solicited adverse events following additional vaccination. Maximum severity of solicited local and systemic reactions over 7 days post-additional (**A-C**) mRNA-1273, (**D-F**) BNT162b2, or (**G-H**) AD26.COV2.S vaccination in participants in the (**A, D,** and **G**) mycophenolate mofetil/mycophenolic acid (MMF/MPA)-, (**B** and **E**) methotrexate (MTX)-, or (**C, F,** and **H**) B cell-depleting therapy (BCDT) cohorts.

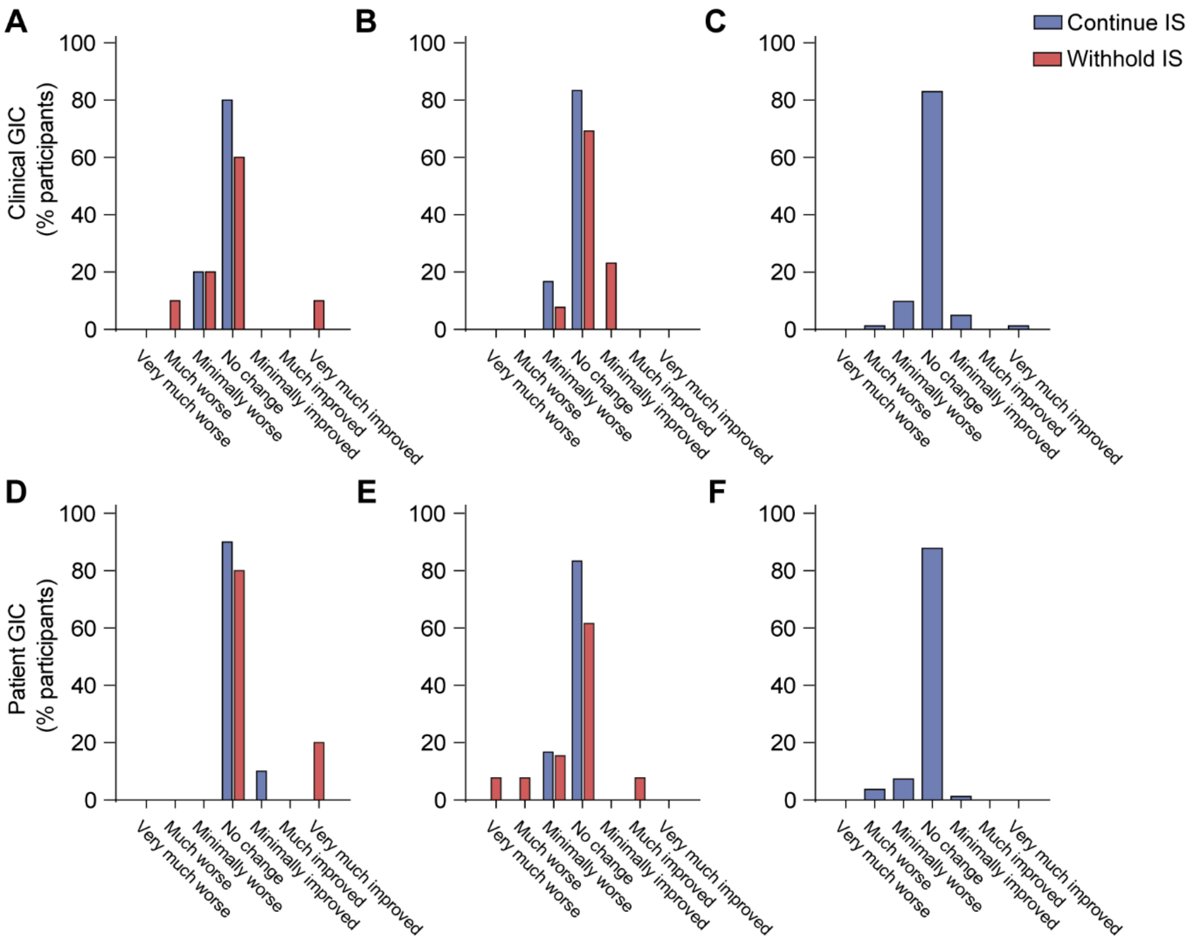

Supplemental Figure 12. Changes in disease activity in the vaccinated population that received a third mRNA vaccine. (**A-C**) Clinical and (**D-F**) patient global impression of change (GIC) at week 4 post-third mRNA vaccine in participants in the (**A** and **D**) mycophenolate mofetil/mycophenolic acid-, (**B** and **E**) methotrexate-, or (**C** and **F**) B cell-depleting therapy-treated cohorts.

Supplemental Table 1. Baseline demographics and characteristics by disease in the vaccinated population.

	SLE (n=27)	RA (n=38)	MS (n=67)	SSc (n=6)	Pemphigus (n=3)
**Sex, n (%)**					
Female	24 (89)	36 (95)	44 (66)	6 (100)	1 (33)
Male	3 (11)	2 (5)	23 (34)	0	2 (67)
**Race, n (%)**					
White	13 (48)	25 (66)	60 (90)	5 (83)	2 (67)
Black	13 (48)	6 (16)	6 (9)	1 (17)	1 (33)
Asian	0 (0)	3 (8)	0 (0)	0 (0)	0 (0)
Native Hawaiian or Other Pacific Islander	0 (0)	0 (0)	0 (0)	0 (0)	0 (0)
American Indian or Alaska Native	0 (0)	1 (3)	0 (0)	0 (0)	0 (0)
Multiple	0 (0)	2 (5)	0 (0)	0 (0)	0 (0)
Unknown	1 (4)	1 (3)	1 (2)	0 (0)	0 (0)
**Ethnicity, n (%)**					
Hispanic or Latino	6 (22)	3 (8)	5 (8)	2 (33)	0 (0)
Not Hispanic or Latino	21 (78)	35 (92)	62 (93)	4 (67)	3 (100)
**Age (years),** mean (SD)	49.1 (15.4)	63.0 (11.7)	45.8 (11.3)	55.8 (15.6)	69.7 (6.8)
**Response to initial COVID-19 vaccination[a],** n (%)					
Negative	9 (33)	17 (45)	48 (72)	3 (50)	2 (67)
Sub-optimal	18 (67)	21 (55)	19 (28)	3 (50)	1 (33)
**Weeks since initial COVID-19 vaccination[b],** mean (SD)	23.1 (8.6)	25.2 (8.1)	25.5 (5.5)	29.8 (5.6)	31.1 (3.1)
**Prior COVID-19 diagnosis,** n (%)	2 (7)	1 (3)	4 (6)	1 (17)	0 (0)
**Vaccine received,** n (%)					
BNT162b2	21 (78)	22 (58)	34 (51)	4 (67)	1 (33)
mRNA-1273	4 (15)	16 (42)	26 (39)	1 (17)	2 (67)
AD26.COV2.S	2 (7)	0 (0)	7 (10)	1 (17)	0 (0)
**MMF-treated**, n	16	1	0	3	1
mg/day, mean (SD)	2375 (806.2)	1000 (-)	NA	1833 (1040.8)	3000 (-)
**MPA-treated**, n	2	0	0	2	0
mg/day, mean (SD)	900.0 (763.7)	NA	NA	2160 (0.0)	NA
**MTX-treated**, n	6	25	0	0	0
mg/week, mean (SD)	21.3 (3.5)	17.1 (5.6)	NA	NA	NA
**Ocrelizumab-treated**, n	0	0	62	0	0
mg/6 months, mean (SD)	NA	NA	585.5 (64.9)	NA	NA
**Ofatumumab-treated**. N	0	0	3	0	0
mg/month, mean (SD)	NA	NA	20.0 (0.0)	NA	NA
**Rituximab-treated**, n	5	18	2	1	2
mg, mean (SD)	1000 (0.0)	972.2 (117.9)	1000 (0.0)	1000 (-)	1000 (0.0)
**Prednisone-treated**, n	14	6	0	0	1
Mg/day, mean (SD)	6.0 (3.4)	4.1 (2.4)	NA	NA	5.0 (-)
**Physician's Global Assessment**, mean (SD)	1.6 (1.8)	1.6 (1.5)	1.5 (2.6)	1.6 (1.0)	0.4 (0.6)
**Patient's Global Assessment**, mean (SD)	4.4 (2.9)	4.2 (2.7)	3.2 (2.7)	4.9 (2.1)	3.5 (3.2)
**SLEDAI**, mean (SD)	3.3 (4.0)	NA	NA	NA	NA
**DAS28-CRP**, mean (SD)	NA	2.9 (1.3)	NA	NA	NA
**EDSS**, mean (SD)	NA	NA	3.0 (2.0)	NA	NA
**mRSS**, mean (SD)	NA	NA	NA	6.2 (10.8)	NA
**PDAI**, mean (SD)	NA	NA	NA	NA	0.3 (0.6)

[a]Negative defined as a Roche Elecsys® Anti-SARS-CoV-2 S result <u><</u>0.79 U/mL and sub-optimal defined as a Roche Elecsys® Anti-SARS-CoV-2 S result >0.79 and ≤ 200 U/mL following the initial COVID-19 vaccine regimen

[b]Defined as weeks since completed initial course of vaccine to informed consent

DAS28-CRP, Disease Activity Score-28 for Rheumatoid Arthritis with CRP; EDSS, Kurtzke Expanded Disability Status Scale; MMF, mycophenolate mofetil; MPA, mycophenolic acid; mRSS, modified Rodnan Skin Score; MS, multiple sclerosis; MTX, methotrexate; PDAI, Pemphigus Disease Area Index; RA, rheumatoid arthritis; SLE, systemic lupus erythematosus; SLEDAI, Systemic Lupus Erythematosus Disease Activity Index; SSc, systemic sclerosis

Supplemental Table 2. Week 4 SARS-CoV-2 antibody concentrations.

GM (%CV)	MMF/MPA Continue (n=10)	MMF/MPA Withhold (n=10)	MTX Continue (n=11)	MTX Withhold (n=12)	BCDT (n=80)
**Spike**	471.36 (555.85)	733.22 (2239.88)	654.50 (734.18)	1858.98 (267.17)	6.41 (6592.25)
**RBD**	453.25 (1552.26)	597.62 (5609.26)	863.31 (527.74)	2671.09 (269.09)	8.21 (1759.38)
**N**	1.52 (143.78)	1.95 (160.07)	2.35 (1485.52)	1.50 (114.23)	0.53 (531.11)

BCDT, B cell-depleting therapy; CV, coefficient of variation; GM, geometric mean; MMF/MPA, mycophenolate mofetil/mycophenolic acid; MTX, methotrexate.
Antibody concentrations (IU/mL) were measured using the V-PLEX SARS-CoV-2 384 Panel 1 IgG kit (Meso Scale Diagnostics, LCC., Rockville, USA).

Supplemental Table 3. Week 4 anti-spike concentrations across SARS-CoV-2 variants.

GM (%CV)	MMF/MPA Continue (N=10)	MMF/MPA Withhold (N=10)	MTX Continue (N=11)	MTX Withhold (N=12)	BCDT (N=80)
**Wuhan-Hu-1**	46220.16 (471.71)	80806.30 (1623.73)	63299.42 (504.55)	161512.40 (250.96)	945.67 (3003.47)
**Alpha**	44030.42 (427.44)	84666.58 (1395.38)	69053.14 (535.72)	146217.12 (248.96)	944.45 (4966.19)
**Beta**	44480.72 (473.49)	109369.62 (939.36)	74430.51 (490.76)	137451.45 (230.63)	837.74 (7434.65)
**Delta**	54489.89 (468.67)	141244.79 (680.69)	85639.34 (487.14)	189333.21 (219.96)	1179.20 (7824.45)
**Omicron**					
BA.1	11820.34 (588.36)	16413.24 (3541.53)	17294.15 (578.75)	43205.81 (272.32)	157.02 (5421.06)
BA.2	17476.90 (559.49)	36210.49 (1504.57)	28322.41 (611.52)	58319.44 (247.09)	360.15 (5849.50)
BA.2.12.1	16423.80 (533.34)	32604.67 (1396.49)	26127.24 (561.35)	53907.62 (248.79)	337.31 (6010.14)
BA.2.75	12225.09 (582.81)	25007.18 (1631.15)	18892.55 (577.65)	41449.28 (260.33)	213.45 (7757.85)
BA.5	16577.41 (531.45)	29866.63 (1431.15)	26307.20 (576.01)	55649.46 (254.49)	288.04 (6122.06)

BCDT, B cell-depleting therapy; CV, coefficient of variation; GM, geometric mean; MMF/MPA, mycophenolate mofetil/mycophenolic acid; MTX, methotrexate.
Antibody concentrations (AU/mL) were measured using the V-PLEX SARS-CoV-2 Key Variant Spike Panel 1 IgG kit (Meso Scale Diagnostics, LCC., Rockville, USA).

Supplemental Table 4. Week 4 neutralization across SARS-CoV-2 variants.

GM(%CV)	MMF/MPA		MTX		BCDT
	Continue (N=10)	Withhold (N=10)	Continue (N=11)	Withhold (N=12)	(N=80)
**Wuhan-Hu-1**	7.17(576.80)	10.12(481.10)	5.98(305.32)	28.04(5,497.08)	1.15(34.70)
**Alpha**	3.92(253.56)	5.11(208.03)	3.59(154.71)	13.74(1,625.84)	1.12(28.52)
**Beta**	2.69(170.54)	2.97(116.05)	2.32(108.70)	4.53(452.08)	1.04(17.01)
**Delta**	4.24(263.20)	4.29(150.26)	3.22(168.55)	11.77(1,236.38)	1.08(25.02)
**Omicron**					
BA.1	1.12(35.82)	1.58(133.41)	1.13(28.60)	2.00(123.76)	1.00(0.00)
BA.2	1.20(40.49)	1.40(70.57)	1.13(28.60)	2.02(128.31)	1.00(0.00)
BA.2.12.1	1.58(63.50)	1.71(81.53)	1.33(55.57)	2.53(169.27)	1.00(0.00)
BA.2.75	1.41(52.11)	1.45(58.78)	1.13(28.60)	1.97(131.42)	1.01(7.76)
BA.5	1.60(55.14)	1.91(80.45)	1.51(55.39)	2.80(145.40)	1.00(0.00)

BCDT, B cell-depleting therapy; CV, coefficient of variation; GM, geometric mean; MMF/MPA, mycophenolate mofetil/mycophenolic acid; MTX, methotrexate.
Neutralization (ACE2 fold inhibition) was measured using the V-PLEX ACE2 Neutralization kit (Meso Scale Diagnostics, LCC., Rockville, USA).

Supplemental Table 5. Baseline associations with week 4 anti-RBD response in the vaccinated population that received an mRNA vaccine.

	MMF/MPA or MTX (n=43)		BCDT (n=80)	
	GM (CV)	p-value[#]	GM (CV)	p-value[a]
**Vaccine Type**				
mRNA-1273	1,510 (515.4)	0.46	9 (943.1)	0.37
BNT162b2	824 (1,312.3)		8 (3,104.6)	
**Immunosuppressant treatment**		0.12		
Withhold	1,352 (1,302.0)		NA	
Continue	635 (835.4)		NA	
**Sex**		0.94		0.8
Male	1,975 (45.1)		7 (1,543.9)	
Female	884 (1,247.1)		9 (1,952.7)	
**Age**		0.61		0.76
≤ Median (51 years)	1,253 (967.0)		7 (1,861.2)	
> Median (51 years)	812 (1,178.3)		10 (1,732.1)	
**Disease Type**		**0.026**		0.47
Systemic Lupus Erythematosus	788 (2,068.6)		4 (6,828.3)	
Rheumatoid Arthritis	2,483 (184.5)		4 (683.2)	
Multiple Sclerosis	-		10 (1,951.0)	
Systemic Sclerosis	73 (148.8)		49 (-)	
Pemphigus	57 (-)		21 (-)	
**Weeks since original COVID-19 vaccination**		0.55		0.44
≤ Median (25.1 weeks)	832 (829.5)		7 (1,033.0)	
> Median (25.1 weeks)	1,017 (1,395.7)		11 (3,320.5)	
**Prior COVID-19**		0.24		0.36
Yes	6,504		17 (1,851.5)	
No	893 (1,079.0)		8 (1,783.2)	
**Prednisone use**		0.67		0.77
Yes	1,585 (240.8)		15 (12,149.3)	
No	725 (2,025.7)		8 (1,530.1)	
**Medication**		0.17		0.23
MMF/MPA	520 (2,494.9)		NA	
MTX	1,556 (417.8)		NA	
Rituximab	NA		5 (1,289.3)	
Ocrelizumab	NA		10 (1,974.2)	
Ofatumumab	NA		23 (3,390.9)	
**Baseline CD19 count**				**0.0014**
Absent	NA		4 (741.1)	
Present	NA		29 (3,204.9)	
**Days since last BCDT dose**				0.13
≤ Median (127 days)	NA		5 (536.4)	
> Median (127 days)	NA		14 (4,566.9)	
**Years since Disease Diagnosis**		1.0		0.94
≤ Median (8.1 years)	1,047 (756.6)		9 (2,231.4)	
> Median (8.1 years)	855 (1,532.5)		8 (1,440.4)	

BCDT, B cell-depleting therapy; CV, coefficient of variation; GM, geometric mean; MMF/MPA, mycophenolate mofetil/mycophenolic acid; MTX, methotrexate

[a]Wilcoxon rank-sum test, bold text denotes statistical significance ($p<0.05$)

Supplemental Table 6. Incidence of adverse events (AEs) in participants who received a third mRNA vaccine.

	MMF/MPA				MTX				BCDT	
	Continue		Withhold		Continue		Withhold			
	Subjects (n=10)	Events	Subjects (n=10)	Events	Subjects (n=13)	Events	Subjects (n=13)	Events	Subjects (n=85)	Events
**AEs**	4 (40)	6	4 (40)	5	5 (39)	7	6 (46)	6	39 (46)	55
Grade 1	4 (40)	5 (83)	3 (30)	3 (60)	1 (8)	1 (14)	2 (15)	2 (33)	19 (22)	20 (36)
Grade 2	1 (10)	1 (17)	1 (10)	2 (40)	4 (31)	6 (86)	3 (23)	3 (50)	20 (24)	22 (40)
Grade 3	0 (0)	0 (0)	0 (0)	0 (0)	0 (0)	0 (0)	1 (8)	1 (17)	10 (12)	13 (24)
Grade 4	0 (0)	0 (0)	0 (0)	0 (0)	0 (0)	0 (0)	0 (0)	0 (0)	0 (0)	0 (0)
Grade 5	0 (0)	0 (0)	0 (0)	0 (0)	0 (0)	0 (0)	0 (0)	0 (0)	0 (0)	0 (0)
Related to Vaccine	0 (0)	0 (0)	0 (0)	0 (0)	0 (0)	0 (0)	0 (0)	0 (0)	6 (7)	6 (11)
**SAEs**	0 (0)	0	0 (0)	0	0 (0)	0	1 (8)	1	9 (11)	11
Related to Vaccine	0 (0)	0 (0)	0 (0)	0 (0)	0 (0)	0 (0)	0 (0)	0 (0)	1 (1)	1 (9)
**MAAE**[a]	0 (0)	0 (0)	0 (0)	0 (0)	0 (0)	0 (0)	0 (0)	0 (0)	1 (1)	1 (2)
**NOCMC**[b]	0 (0)	0 (0)	0 (0)	0 (0)	0 (0)	0 (0)	0 (0)	0 (0)	7 (8)	7 (13)
**AESI**										
Myocarditis	0 (0)	0 (0)	0 (0)	0 (0)	0 (0)	0 (0)	0 (0)	0 (0)	0 (0)	0 (0)
Pericarditis	0 (0)	0 (0)	0 (0)	0 (0)	0 (0)	0 (0)	0 (0)	0 (0)	0 (0)	0 (0)

Values depict n (%)
Percentages for the number of participants with events are based on the number of participants in the vaccinated population.
AE, adverse event; AESI, adverse events of special interest; MAAE, medically attended adverse event; NOCMC, new-onset chronic medical condition; SAE, serious adverse event
[a]MAAE defined as a hospitalization, emergency room visit, or unscheduled medical visit for any reason and considered related to the vaccine. [b]NOCMC is defined as any new ICD diagnosis during the study after receipt of the vaccine that will continue for at least 3 months and requires continued health care intervention.

Supplemental Table 7. Incidence of adverse events (AEs) in participants who received a second AD26.COV2.S vaccine.

	**MMF/MPA Withhold**		**BCDT**	
	Subjects (n=2)	Events	Subjects (n=8)	Events
**AEs**	1 (50)	1	6 (75)	6
Grade 1	0 (0)	0 (0)	4 (50)	4 (67)
Grade 2	0 (0)	0 (0)	2 (25)	2 (33)
Grade 3	1 (100)	1 (100)	0 (0)	0 (0)
Grade 4	0 (0)	0 (0)	0 (0)	0 (0)
Grade 5	0 (0)	0 (0)	0 (0)	0 (0)
Related to Vaccine	0 (0)	0 (0)	0 (0)	0 (0)
**SAEs**	1 (50)	1	0 (0)	0
Related to Vaccine	0 (0)	0 (0)	0 (0)	0 (0)
**MAAE**[a]	0 (0)	0 (0)	0 (0)	0 (0)
**NOCMC**[b]	0 (0)	0 (0)	0 (0)	0 (0)
**AESI**				
Myocarditis	0 (0)	0 (0)	0 (0)	0 (0)
Pericarditis	0 (0)	0 (0)	0 (0)	0 (0)

Values depict n (%)
Percentages for the number of participants with events are based on the number of participants in the vaccinated population.
AE, adverse event; AESI, adverse events of special interest; MAAE, medically attended adverse event; NOCMC, new-onset chronic medical condition; SAE, serious adverse event; VAE, vaccine-related adverse event
[a]MAAE defined as a hospitalization, emergency room visit, or unscheduled medical visit for any reason and considered related to the vaccine.
[b]NOCMC is defined as any new ICD diagnosis during the study after receipt of the vaccine that will continue for at least 3 months and requires continued health care intervention.

Supplemental Table 8. Disease flares at 4 weeks post-booster vaccination in participants who received a third mRNA vaccine.

	**MMF/MPA**		**MTX**		**BCDT**	**All**
	Continue	Withhold	Continue	Withhold		
**SLE participants**, n	7	8	3	3	4	25
Severe flare[a], n (%)	0 (0)	0 (0)	0 (0)	0 (0)	0 (0)	0 (0)
Mild/moderate flare, n (%)	3 (43)	1 (13)	1 (33)	0 (0)	0 (0)	5 (20)
**RA participants**, n	0	0	9	10	17	36
Flare[b], n (%)	NA	NA	0 (0)	0 (0)	2 (12)	2 (6)
**MS participants**, n	0	0	0	0	60	60
Relapse[c], n (%)	NA	NA	NA	NA	0 (0)	0 (0)
**SSc participants**, n	3	1	0	0	1	5
Severe flare[d], n (%)	0 (0)	0 (0)	NA	NA	0 (0)	0 (0)
**Pemphigus participants**, n	0	1	0	0	2	3
Severe flare[e], n (%)	NA	1 (100)	NA	NA	0 (0)	1 (33)

BCDT, B cell-depleting therapy; MMF/MPA, mycophenolate mofetil/mycophenolic acid; MTX, methotrexate; MS, multiple sclerosis; RA, rheumatoid arthritis; SSc, systemic sclerosis; SLE, systemic lupus erythematosus

Disease activity measured by [a]Thanou modified SELENA-SLEDAI Flare Index; [b]increase in DAS-28 score >1.2 or >0.6 if the Week 4 DAS-28 score is >3.2; [c]Physician-assessed relapse for MS; [d]onset of new or significant worsening of internal organ involvement requiring hospitalization or change in treatment or worsening of skin thickening on the modified Rodnan Skin Score of >4 units; [e]At least 3 new lesions a month that do not heal spontaneously within 1 week or by the extension of established lesions in a participant who has achieved disease control

Supplemental Table 9. Participating study centers

Site Name	Site PI	Screened	Randomized/ Allocated
Benaroya Research Institute	Sandra Lord, MD	4	4
Brigham and Women's Hospital	Jeffrey A. Sparks, MD, MMSc	12	8
Cleveland Clinic	Jeffrey Cohen, MD	19	11
Columbia University	Anca Askanase, MD	10	0
Duke University Medical Center	Ankoor Shah, MD	1	0
Emory University	Arezou Khosroshahi, MD	42	10
Feinstein Institute for Medical Research	Meggan Mackay, MD	42	34
Massachusetts General Hospital – Division of Rheumatology	Zachary Wallace, MD	18	11
Michigan Medicine	Dinesh Khanna, MD	29	19
Medical University of South Carolina	Diane L. Kamen, MD	9	1
New York University Langone Orthopedic Center	Amit Saxena, MD	10	4
Oklahoma Medical Research Foundation	Judith James, MD, PhD	47	25
Temple University, Lewis Katz SOM	Roberto Caricchio, MD	5	4
University of California, Los Angeles Division of Rheumatology	Maureen McMahon, MD	0	0
University of Pennsylvania Perelman Center for Advanced Medicine	Amit Bar-Or, MD	26	14
University of Rochester Medical Center	Christopher Palma, MD, ScM	0	0
University of Texas Health Science Center at Houston	Maureen D. Mayes, MD	0	0
Washington University School of Medicine	Alfred Kim, MD, PhD	3	2
Yale University School of Medicine	Fotios Koumpouras, MD	2	1

Supplemental Acknowledgements

ACV01 Study Team	Institution
Alise Carlson, MD	Cleveland Clinic
Deja Rose, MD	Cleveland Clinic
Lindsay Ross, MD	Cleveland Clinic
Sarah Simmons, MD	Cleveland Clinic
Ahmad Mahadeen, MD	Cleveland Clinic
Megan Elder, RN BSN	Cleveland Clinic
Leah Tardivo, RN BSN	Cleveland Clinic
Bryan Davies, RN	Cleveland Clinic
Diane Ivancic	Cleveland Clinic
Stephanie Moore	Cleveland Clinic
Sadie Coates	Cleveland Clinic
Michelle Maxson	Cleveland Clinic
Rachael Yim	Cleveland Clinic
Jenna Titus	Cleveland Clinic
Ariel S. Bizon	Cleveland Clinic
Alecia Chase, RN BSN	Cleveland Clinic
Moein Amin, MD	Cleveland Clinic
Lisa Stropp, MD	Cleveland Clinic
Jameson Holloman, MD	Cleveland Clinic
Megan Roser	Cleveland Clinic
Jacqueline Dinishak	Cleveland Clinic
Amanda Evans	Cleveland Clinic
Thomai Skaramagas	Cleveland Clinic
Cristina Arriens, MD	Oklahoma Medical Research Foundation
Teresa Aberle, PA-C	Oklahoma Medical Research Foundation
Kelli Kraus	Oklahoma Medical Research Foundation
Micki Drake	Oklahoma Medical Research Foundation
Jason L. Larabee	University of Oklahoma Health Sciences
Patricia Gonzales, LVN	University of Texas Health Science Center
Julio Charles, MS	University of Texas Health Science Center
Asaff Harel, MD	Northwell Health
Erik Anderson, MD, PhD	Northwell Health
Cynthia Aranow, MD	Northwell Health
Janine Sullivan, NP	NYU Langone Health
Monica Gamez-Perez, RN	NYU Langone Health
Emilie Schurenberg	NYU Langone Health
Abraham Sinay-Smith	NYU Langone Health
